# Discovering Conformational Sub-States Relevant to Protein Function

**DOI:** 10.1371/journal.pone.0015827

**Published:** 2011-01-28

**Authors:** Arvind Ramanathan, Andrej J. Savol, Christopher J. Langmead, Pratul K. Agarwal, Chakra S. Chennubhotla

**Affiliations:** 1 Computational Biology Institute and Computer Science and Mathematics Division, Oak Ridge National Laboratory, Oak Ridge, Tennessee, United States of America; 2 Department of Computational and Systems Biology, University of Pittsburgh, Pittsburgh, Pennsylvania, United States of America; 3 Joint Carnegie Mellon University–University of Pittsburgh Ph.D. Program in Computational Biology, Pittsburgh, Pennsylvania, United States of America; 4 Computer Science Department, School of Computer Science, Carnegie Mellon University, Pittsburgh, Pennsylvania, United States of America; 5 Lane Center for Computational Biology, School of Computer Science, Carnegie Mellon University, Pittsburgh, Pennsylvania, United States of America; University of Milano-Bicocca, Italy

## Abstract

**Background:**

Internal motions enable proteins to explore a range of conformations, even in the vicinity of native state. The role of conformational fluctuations in the designated function of a protein is widely debated. Emerging evidence suggests that sub-groups within the range of conformations (or sub-states) contain properties that may be functionally relevant. However, low populations in these sub-states and the transient nature of conformational transitions between these sub-states present significant challenges for their identification and characterization.

**Methods and Findings:**

To overcome these challenges we have developed a new computational technique, quasi-anharmonic analysis (QAA). QAA utilizes higher-order statistics of protein motions to identify sub-states in the conformational landscape. Further, the focus on anharmonicity allows identification of conformational fluctuations that enable transitions between sub-states. QAA applied to equilibrium simulations of human ubiquitin and T4 lysozyme reveals functionally relevant sub-states and protein motions involved in molecular recognition. In combination with a reaction pathway sampling method, QAA characterizes conformational sub-states associated with cis/trans peptidyl-prolyl isomerization catalyzed by the enzyme cyclophilin A. In these three proteins, QAA allows identification of conformational sub-states, with critical structural and dynamical features relevant to protein function.

**Conclusions:**

Overall, QAA provides a novel framework to intuitively understand the biophysical basis of conformational diversity and its relevance to protein function.

## Introduction

Proteins are not static entities, but exist as a dynamic ensemble of inter-converting conformations. These ensembles exhibit a wide range of spatial and temporal scales of internal motions; localized protein motions involving bond vibrations and fluctuations within a group of few atoms are fast (femtosecond-picosecond time-scale) where as large-scale concerted, collective fluctuations involving sub-domains or entire protein are typically slow (millisecond time-scale and beyond) [1]–[3]. These wide range of motions show inter-dependency, leading to a highly complex organization of the conformational and energetic landscape [4]. Several studies have shown that the protein's conformational and energetic landscape is organized in a multi-level hierarchy [5]–[8].

In the familiar representation, one can imagine the potential energy landscape to be rugged and be formed of *hills* and *valleys* of varying heights and depths, populated by conformations of the protein. Within each valley, the population of conformations share significant similarity in terms of their structures as well as internal energies. The sub-population of protein conformations within each of these valleys represent a *sub-state*. The multiple levels in the hierarchy stem from the energetic differences (and energy barriers) between the various sub-states. Internal protein motions driven by thermodynamical energy fluctuations allow the protein to transition from one sub-state to another. In cases where several sub-states are separated by small energy barriers from each other but collectively by a larger barrier from other sub-states, together the collection of these sub-states can be viewed as a new sub-state in the multi-level hierarchy.

Internal protein motions correspond to the inter-conversion of protein conformations as they move within a sub-state or as they visit from one sub-state to another [9], [10]. Analyses of internal protein motions based on experimental and theoretical/computational approaches have established the importance of sampling multiple sub-states as being vital for a number of protein functions including molecular recognition [11], enzyme catalysis [9] and allosteric modulation [12]. A number of enzymes have attracted considerable interest due to the connection between conformational fluctuations and the catalytic mechanisms [3], [13]–[15]. An intriguing observation has been that large conformation fluctuations occur in distal regions of the protein, far away (>10 Å) from the active-site, which influence the catalytic step [14]–[19]. However, it is not known if these distal motions are somehow related to the ability of enzymes to sample conformations that facilitates the attainment of the transition state during the reaction mechanism. More recently, fascinating insights from X-ray crystallographic studies have indicated that there may be rare (*hidden*) conformations and sub-states that critically alter the active site environment for catalysis [20]. Internal motions have also been implicated in biomolecular recognition by proteins [11], [21], [22]. Hence, apart from implicating the flexibility of a protein, it is also equally critical to elucidate possible conformational sub-states (including the ones with low-probabilities) and the structural changes that enable the protein to explore these sub-states.

Experimental techniques revealed a wealth of information about the inter-connection between conformational fluctuations and protein function. X-ray studies and nuclear magnetic resonance (NMR) methods have provided information about the most populated states (or conformational sub-states) for an increasing number of proteins [23], [24]. Further, pioneering work of Hammes and co-workers have provided information about conformations associated with single molecules during enzyme catalysis [25]. Recently, enzyme cyclophilin A has been investigated extensively for connection between protein dynamics and enzyme catalysis. NMR spin relaxation studies performed by Kern and coworkers linked the motions of several residues with the substrate turnover step in cyclophilin A, and also indicated that the rate of enzyme conformational changes coincides with the rate-limiting step of substrate turnover [14], [20], [26]. NMR studies by Lange and co-workers have provided insights into the structural heterogeneity of ubiquitin, relevant to its function of binding multiple proteins, at the 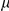
s time-scales [11]. Even though surface regions of ubiquitin and their collective motions have been implicated in binding, the conformational sub-states involved in the mechanism of molecular recognition have been difficult to characterize. Similarly, correlated motions have been implicated in sub-domain motions for lysozyme [27], [28]. The detailed characterization of how these motions lead the protein to sample specific sub-states is yet unknown. The experimental techniques tend to provide ensemble averaged information and are limited to probing dynamics within narrow windows of time-scales, depending on the instrument resolution.

Computational simulations allow bridging multiple time-scales and provide detailed atomistic insights into protein motions [13], [22], [29]–[31]. Agarwal and co-workers performed computational studies of cyclophilin A and identified a network of protein residues whose motions influenced the reactive trajectories in the active-site [18], [19], [32]. For ubiquitin, flexibility at 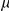
s time-scales have provided some insights into the conformational diversity of how ubiquitin may recognize its binding partners [22]. Similar insights are also available for lysozyme [33], [34] from atomistic simulations; however, it is unclear if these motions translate into transitions between sub-states. Therefore, it would be ideal to simultaneously characterize both the flexibility of the protein and possible transitions enabled by the protein's flexibility between sub-states that are functionally relevant. The achievable time-scales of computational simulations continue to slowly reach towards biologically relevant time-scales. The large number of conformations sampled during single or multiple molecular dynamics (MD) simulations poses a challenge for analysis.

Computational tools to analyze and identify conformational sub-states in the multi-level hierarchy that will enable to intuitively understand the biophysical basis of conformational diversity and its relevance to protein function are still limited. The conformations sampled during MD simulations correspond to a highly multi-dimensional data set due to the large number of degrees of freedom associated with the protein. Characterizing the high-dimensional multi-variate data, which is embodied in these MD simulations, is a long standing problem in statistics and related fields [35]. Indeed, descriptions of the conformational landscapes spanned by protein motions have typically relied on finding motion directions that can provide biophysically meaningful interpretations. Note, we realize that the conformational ensemble can be projected onto low dimensional representations based on a variety of methods. However, the challenge lies in identifying groups of conformations (sub-states) that provide new insights into the mechanism of protein function.

QAA is based on pursuing higher order statistics of positional deviations associated with the conformational data sampled during the MD simulations. Using three different proteins - human ubiquitin, T4 lysozyme and enzyme cyclophilin A - we show that QAA identifies and characterizes the conformational sub-states relevant to function. Based on the inspection of the conformation populations in the sub-states using parameters such as internal energy or other biophysically relevant order parameters, we observe that the identified sub-states contain crucial structural and dynamical elements relevant to promoting the designated function of each of these proteins. A recursive application of QAA yields a multi-level motion hierarchy with global modes dominating the top level and subsequent levels revealing progressively localized motions within the proteins. Additionally, the rare-conformational transitions associated with the interconversion between the identified sub-states allows vital insights into these protein's structure, motions and function.

## Results

This section is organized as follows. First, the conformational diversity observed in computational (MD) simulations is examined for anharmonic motions. Then, the theoretical details behind QAA are presented. Finally, QAA is illustrated on three different model systems: (1) human ubiquitin, (2) T4 lysozyme, and (3) enzyme human cyclophilin A. The results provide insights into how intrinsic fluctuations in each of these proteins enable the functionally important conformations to be sampled. In the discussion section, we compare QAA with other computational techniques that are also used to characterize the conformational diversity.

### Quantifying anharmonicity in protein fluctuations

A common measure for exploring anharmonicity (or non-Gaussianity) is the fourth-order statistic kurtosis, 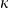, defined for a random variable 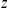 as the normalized fourth central moment:
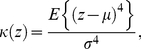
(1)where 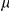 is the mean and 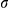 the standard deviation of 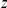, and 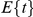 denotes the expected value of the quantity 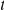. For unimodal distributions, kurtosis is a means of quantifying their peakiness or equivalently the proportion of the weight in the tails. A Gaussian distribution with zero-mean and unit variance has 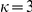. A value of 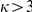 indicates a super-Gaussian distribution (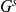) that is more peaked and heavier tailed than the baseline Gaussian (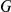). Conversely, a distribution that is less peaked (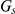) than the baseline Gaussian (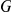) has kurtosis 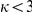. We will use 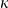 as a measure to quantify the anharmonicity in atomic fluctuations.

Human ubiquitin is used as a prototypical example to examine the nature of atomic fluctuations. For comparison, we use 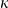 to study the anharmonicity observed in ubiquitin motions from 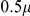
s long MD simulation [22](see Materials and Methods section) and also from experimental ensembles (116 NMR structures revealing up to 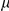
s dynamics [Protein Data Bank (PDB) code: 2K39] [11], and 44 X-ray crystallographic structures).

**Figure 1:**
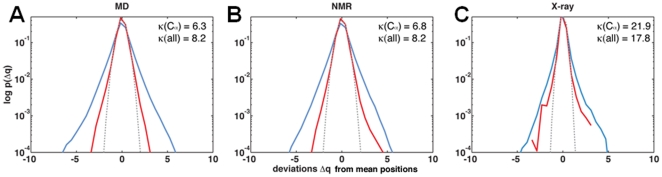
Anharmonic distribution of positional deviations (Å) in ubiquitin from 0.5 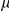
s-MD, NMR, and X-ray ensembles. For each atom, the positional displacement (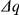) from the mean position was calculated at 50 ps intervals. The same bin size (0.54 Å) was used for all histograms. Dotted curve shows a Gaussian fit to the C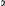 distribution. The probability distributions of positional deviations [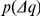] are plotted in log-scale.

In Figure 1A, observe that both 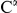 (backbone) and all-atom positional deviations are anharmonic for long-timescale MD data (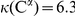; 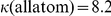), though anharmonicity is observed even at shorter time-scales (Figure S1). Side-chains contribute more to anharmonicity in the protein than C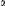 atoms as seen in Figure 1 (blue lines) since side-chains (especially solvent exposed) have greater degree of freedom associated with their motions. Interestingly, both MD and NMR ensembles (Figures 1B and C) show similar anharmonic behavior, although the X-ray ensemble shows higher peakiness and insufficient sampling in regions far from the mean.

**Figure 2:**
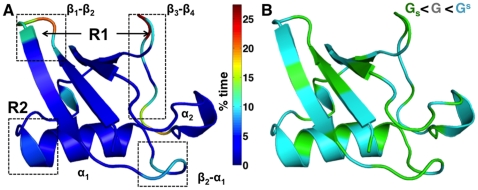
Rare-conformations in ubiquitin are functionally relevant. (A) shows the amount of time spent by each C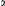 atom exhibiting anharmonic fluctuations. Note that functionally relevant regions in ubiquitin forming primary (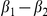 and 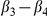 called R1 collectively) and secondary binding interfaces (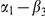 called R2 and 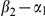) spend relatively a large fraction of the time exhibiting anharmonic fluctuations. (B) illustrates which regions of the protein exhibit 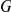 (Gaussian), 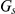 (sub-Gaussian) and 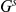 (super-Gaussian) motions. Note that the protein is predominantly anharmonic.

Using a Gaussian fit to the 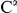 positional deviations from MD simulations, we compute how often each 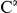 atom is found three standard deviations or more away from the mean of the approximating Gaussian distribution (Figure 2). Ubiquitin's flexible loop regions 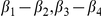 (collectively referred to as region R1), 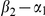, 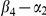, and the C-terminal tip of 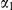 (region R2) of ubiquitin populate the long-tails of the distributions (Figure 2A). Long-tails refer to non-trivial populations at extreme positional deviations. Given that these are highly flexible regions and have a functional role in substrate binding [11], their associated anharmonic distributions warrant closer study. To this end, we examine the kurtosis of the positional deviations projected onto a principal coordinate system built locally for each 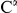 (Figure 2B). We observe that at least 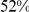 of the 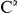 atoms are super-Gaussian (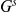; 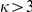) and 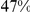 are sub-Gaussian (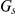; 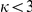) along the first principal components (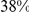 are super-Gaussian along all three principal components). It is further interesting to note that non-Gaussian (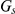 and 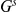) distributions are associated with protein regions R1 and R2, which are both involved in forming primary contacts with substrates [11]. Thus, atomic deviations at functionally relevant protein regions are mixtures of 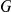, 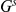, and 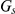 distributions.

**Figure 3:**
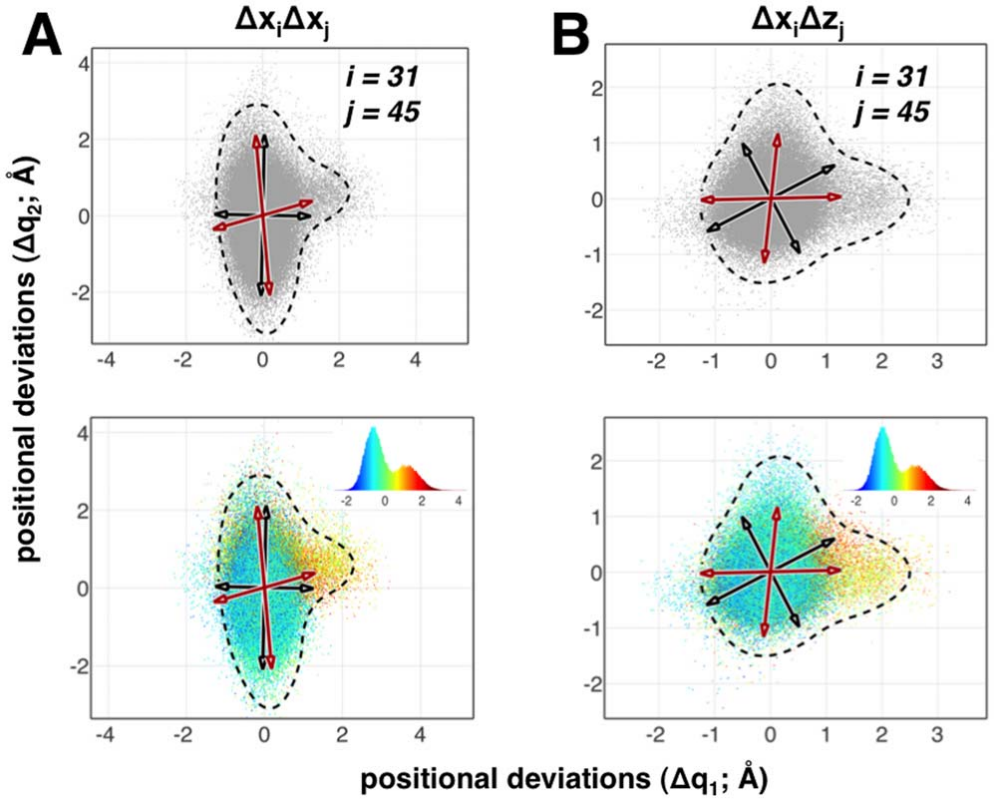
Intrinsic non-orthogonality and energetic coupling in pair-wise distributions of positional fluctuations in ubiquitin. Top panels show pair-wise distributions of atomic fluctuations considered along the C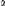 atom's 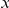 and 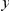 (A) and 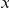 and 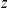 (B) directions for the residue pair 31 and 45. The black arrows represent the directions from QHA whereas the red arrows represent the directions from QAA. Note that only the non-orthogonal QAA directions align well with the natural orientation of the data. QHA directions, which are orthogonal, do not model this distribution well. Lower panels illustrate the energetic coupling in pair-wise distributions. QAA directions are biophysically relevant as they point to directions where the high-energy states exist. The corresponding energy distributions of the pair-wise interactions (non-bonded electrostatic and van der Waals) are shown in respective insets. Although illustrated for a particular residue pair, a thorough comparison of positional fluctuations also reveals that this intrinsic non-orthogonality occurs throughout the protein. The dotted line in these plots represent the extent of these joint distributions, highlighting the anharmonicity in positional fluctuations for the residues considered here.

Individual atoms exhibit significantly anharmonic positional deviations. However, to understand coupling between different protein regions, we examine the joint positional deviations of atom pairs and measure for comparison how a well known approach in the literature, called quasi-harmonic analysis (QHA) [36], models the underlying distributions (Figure 3). When the deviations are more Gaussian-like, the QHA basis vectors, which maximize variance, align well with the intrinsic orientation of the data (Figure S2 and description in Text S1). However, when the source distributions combine 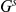 or 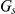, the intrinsic orientations of the data can be non-orthogonal, necessitating *higher-order correlations*. Under these circumstances, QHA does not capture the intrinsic motions in its sole pursuit of variance. Thus, for internal motions of the complete protein (involving 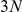 dimensions, where 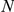 refers to the number of protein atoms), QHA bases may not adequately capture the complex dependencies in positional deviations arising from mixtures of 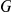, 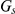, and 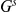 distributions.

From a biophysical perspective, the joint distributions in positional deviations illustrate a potential and more serious limitation of QHA. Considering the same residues illustrated in Figure 3, we paint the positional deviations with the internal energy (sum of van der Waals and electrostatic interactions computed by NAMDEnergy [37]) for each pair of residues considered (Figures 3A and 3B, lower panels). When we examine the case where source distributions combine both 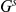 and 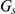, the peripheral regions along the joint positional deviations are enriched by high energy conformers. These peripheral regions represent sub-states that have lower populations, where the motion of one residue implicates a preferential energy state (either low or high) for not only the pair of residues considered, but also for the entire protein (data not shown). The QHA bases (shown as black arrows) poorly align with directions that indicate high-energy states.

### Quasi-anharmonic representation of protein dynamics

To address the issues of both higher-order correlations and non-orthogonality, as well as address the limitations of QHA, we propose *quasi-anharmonic analysis (QAA)*, a method based on diagonalizing a tensor of fourth-order statistics describing positional fluctuations and their couplings. We use an efficient algebraic technique called joint-diagonalization of cumulant matrices (JADE), a well known algorithm in the machine learning literature for analysis of multi-variate data [38].

We model the observable positional deviation vector, 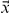, as a linear combination of anharmonic sources, 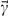, such that: 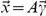. Here, 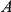 is an unknown coupling matrix where each column 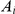 encodes an anharmonic mode of motion describing the intrinsic higher-order correlations between different regions of the protein. The excitation of the anharmonic modes can be quantified as 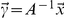. Unlike in QHA, the basis matrix 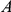 can be non-orthogonal and hence the anharmonic modes can be intrinsically coupled. It is important to estimate both 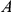 and 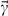 to suitably describe the anharmonic landsacpe. We term this analysis *quasi-anharmonic* for two reasons: first, we study anharmonicity explicitly whereby the sources are fully decorrelated and higher-order dependencies are minimized; second, we impose a linear model which ignores any non-linear coupling that may exist in the fluctuations between different parts of a protein.

To derive 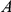 it is instructive to consider QHA, where the positional deviations 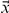 are modeled as a linear combination of harmonic sources 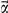 given by
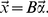
(2)


The harmonic modes 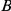 are conveniently expressed by the eigenvalues 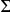 and eigenvectors 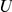 of the covariance matrix given by
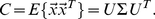
(3)


For exposition, we will set the QHA bases 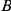 to
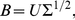
(4)and it follows that
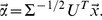
(5)


The covariance matrix 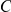 captures only second-order correlations in atomic fluctuations 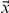 and the QHA basis remove these dependencies, i.e.
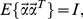
(6)where 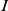 is an identity matrix of size 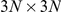. However, 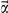 might exhibit higher-order dependencies and we capture this by estimating a fourth order cumulant tensor.

The fourth order cumulant tensor 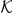 comprises of auto and cross-cumulants given by
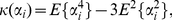
(7)and
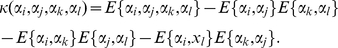
(8)


Since 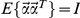, it implies that 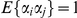 when 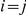 and 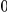 when 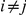. The cumulant tensor will have a total 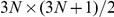 matrices each of size 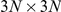 accounting for auto- and cross-cumulant terms.

We can reduce the fourth order dependencies by minimizing the sum of the cross-cumulant terms, which is equivalent to diagonalizing the tensor 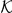. However, no closed form solution exists for diagonalizing a tensor, but an approximate solution can be found using efficient algebraic techniques such as Jacobi rotations [39]. Just as the rotation matrix 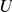 diagonalizes the covariance matrix 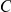, a rotation matrix 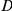 can be found which approximately diagonalizes the cumulant tensor 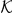, leading to:
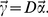
(9)


Substituting for 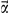 from above:
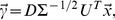
(10)and thus 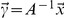 implying
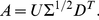
(11)


Thus, 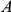 represents the anharmonic modes of motion derived by minimizing the fourth-order dependencies in positional fluctuations, in addition to eliminating the second-order correlations (as is the case with QHA). The anharmonic modes of motion 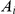, which are the columns of matrix 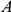, are sorted in decreasing order of their amplitudes (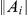). A public domain implementation of the JADE procedure is available in [38].

We first illustrate that QAA works correctly in the pairwise distributions considered in Figure 3. The red arrows in each case show the QAA basis vectors. Observe that when the fluctuations are anharmonic, QAA clearly aligns along the directions which are descriptive of the individual atomic fluctuations. From a biophysical perspective, the QAA directions have important implications for understanding the energy landscape in these two dimensional plots (Figure 3 lower panels and Figure S2 in Text S1). First, note that the alignment along preferential directions of fluctuations in the atoms indicates that QAA can identify and characterize conformational sub-states with low populations in the landscape. Second, the motions described along QAA basis vectors are more relevant to the intrinsic motions of atom-pairs since the directionality of the motions lead to an energetically homogeneous state. This unique ability to distinguish energetically homogenous sub-states enables QAA to provide novel insights into the conformational landscape of the entire protein. These aspects are further elaborated on three model protein systems as described in the subsequent sections.

### Examining the multi-scale conformational diversity in ubiquitin binding using QAA

Ubiquitin is universally expressed in eukaryotes and plays a fundamental role in the proteosomal degradation pathway by labeling specific proteins. The protein's three-dimensional structure is highly conserved over evolution [40]. Further, it is known to bind a large number of proteins with high specificity implying that its intrinsic mechanism of binding is finely tuned to respond to its diverse set of targets. Recently, it was proposed that the solution structure of ligand-free ubiquitin exhibits all (or most) of its conformational diversity required to bind diverse targets [11]. These studies imply that ligand-free ubiquitin might occasionally visit conformations that resemble the ligand-bound structure. Hence, it is of interest to quantify from an ensemble, how many of these conformations exhibit the required diversity to resemble ligand-bound conformations.

**Figure 4:**
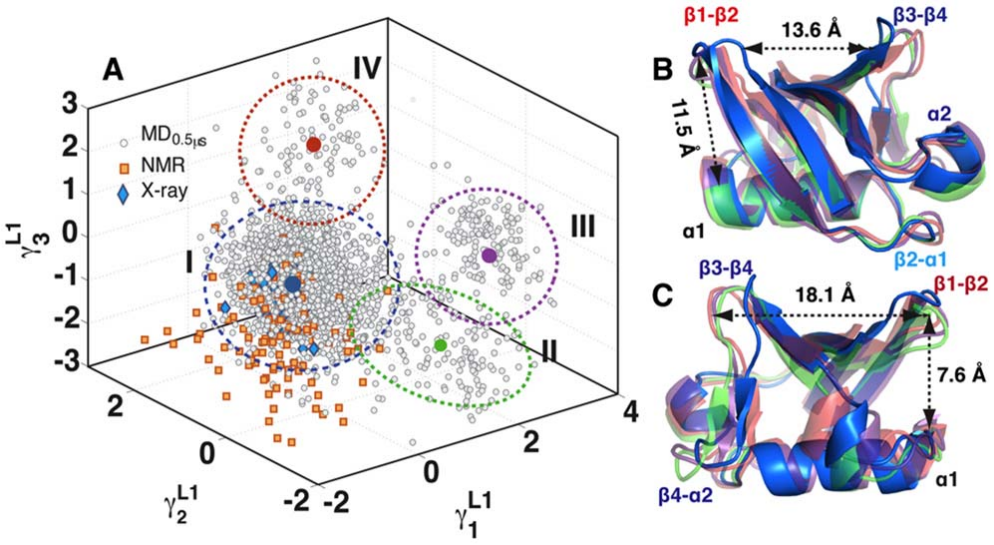
Quasi-anharmonic analysis (QAA) of ubiquitin conformational landscape. (A) The MD ensemble projected onto the top three anharmonic modes of motion. The anharmonic modes are represented by 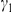, 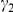 and 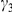. Level 1 (L1) indicates the level of the hierarchy. The projection (units Å) shows four distinct clusters (I-IV). The clusters were identified using a mixture of Gaussian (MoG) [41] model, with boundaries marked by ellipses drawn 3 standard deviations (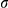) from the respective cluster centers. The cluster centers are shown in blue (7,880 conformers; I) green (773; II), purple (692; III) and red (655; IV). The X-ray ensemble consisting of 44 crystal structures is shown as blue diamonds; 42 of these structures are covered within 2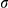 of cluster I. The 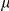
s time-scale NMR ensemble [11] consisting of 116 conformers are shown as orange squares; 78 conformers lie with 3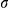 deviations from cluster I, indicating that the MD sampling has visited most bound/unbound conformations in the space spanned by 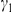, 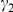 and 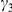. (B and C). Two different view-points (rotated around y-axis by 180°) of the mean conformations from each cluster (bold circles in A) show significant structural deviations in R1 and R2. The distance between centroids of R1 and R2 are shown here for reference. In cluster I, the average distance between R1 is only 13.6 Å where as in the other three clusters (II, III and IV), the distance is 18.1 Å. The distance between R1 and R2 is maximum in cluster I (11.5 Å), where as decreases to about 7.5 Å in clusters II, III and IV.

Here, we considered the 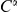 atoms for residues 2-70 (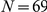) and sampled 10,000 conformations spread evenly over 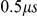 MD. The highly flexible free-ends of ubiquitin (residues 1 and 71-76) were excluded from analysis. The 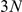 dimensional space was first projected on to the top 30 QHA dimensions (covering 96% of the overall variance). The projection on to this sub-space mitigates the effects of fast and local fluctuations (noise) and provides a subspace tractable for convergence. Projecting the 10,000 conformers of the simulations onto the top three anharmonic modes (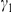, 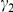 and 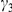), as shown in Figure 4, we observe that the landscape separates into unique conformational wells. Using a mixture-of-Gaussian (MoG) [41] model (for which a public domain implementation is available [42]), we identify four clusters representing conformational wells (labeled I through IV) with boundaries marked by ellipses drawn 3 standard deviations (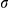) from the respective cluster centers. The mean structures from each well reveal novel features of ubiquitin's ability to sample a wide range of conformations even at equilibrium. In the cluster shown in blue (Figures 4B & 4C) and consisting of over 8,000 structures, ubiquitin adopts a conformation whereby region R1 is constrained (13.6 Å), whereas 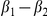 and R2 are far apart (11.5 Å). Observe that a majority of the NMR ensemble (43 conformers within 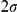 and 78 within 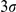) and the X-ray ensemble (42 within 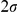 and 44 within 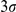) fall within cluster I, indicating that MD sampling has indeed visited all of the bound/unbound conformers observed in this three-dimensional space. QAA reveals three other clusters (shown in purple, green, and red in Figure 4A). They form the peripheral regions of cluster I, exhibiting motions along 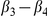 and 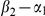 regions, indicating motions complementary to R1 and R2 (Figure 4B and Figure 4C). In cluster IV, the mean structure shows an open conformation where region R1 is extended over 18 Å and R2 is close to 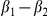 at 7.6 Å. Note that motions in both R1 and R2 are implicated in binding diverse substrates [11], [22], [43].

**Figure 5:**
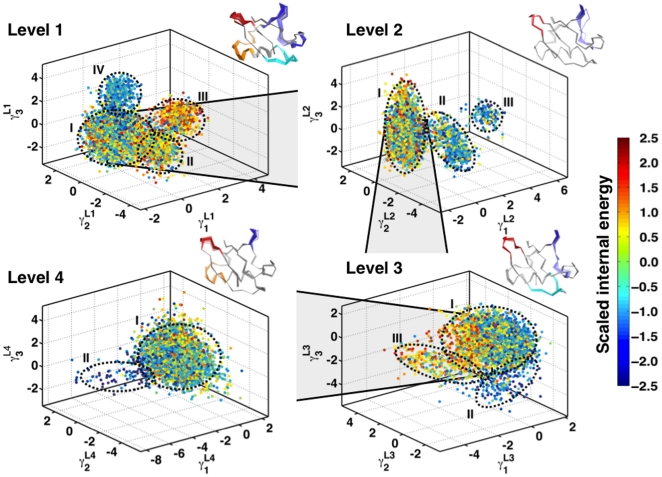
Hierarchical organization of conformational sub-states in ubiquitin motions. Level 1 decomposition identifies four sub-states. Each conformation is colored using the scaled internal energy [44]. The internal energy is the sum of the non-bonded interaction energy between all pairs of residues in the protein. The energy distribution is normalized to be zero-mean, unit-variance for ease of interpretation. Levels 2, 3 and 4 are derived from the largest sub-state of the preceding level indicating more homogeneity in both positional deviations and internal energy. Motions along the top anharmonic mode (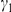) are illustrated in each panel in a movie like representation, showing only the C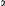 trace of the protein (see SI Movies). The primary and secondary binding regions 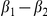, 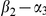, 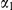 and 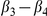 are highlighted in red, cyan, orange and blue respectively to highlight large-scale fluctuations in these regions. While in Level 1 the motions are global - involving the entire protein, Levels 2, 3 and 4 show subsequent localization of motions, as evidenced by their relative decrease in amplitude. The motions in each level involve well defined transitions from a relatively heterogenous population to a energetically homogenous sub-state. These motions have implications in ubiquitin recognizing multiple binding partners [11], [22].

We next examine if these conformational wells exhibit any similarity in terms of their internal energies, defined as the sum of van der Waals and electrostatic energy over all interactions in the protein and computed using the program NAMDEnergy [37]. We plot the scaled internal energy values [44], [45] on the data in Figure 4 and illustrate it in Figure 5 (Level 1). Scaled internal energy refers to the sum of non-bonded interaction (electrostatic and van der Waals) energies between all residues in the protein that have been normalized (zero mean, unit variance). While cluster I shows considerable diversity in its internal energies, clusters II, III and IV are homogeneous. The homogeneity in the internal energy distributions are quantified further in Figure S3 and supporting Text S1. Clusters I and III are separated by high-energy structures possibly indicating a transition state between the two wells. The largest conformational well (cluster I) is highly diverse with respect to its internal energy distributions and positional deviations (Figure 5). Thus, we can examine the conformational diversity in this cluster by iteratively performing QAA only for this subset of conformations to see if a subsequent decomposition might homogenize this landscape. This corresponds to Level 2 in the conformational hierarchy. Figure 5 (Level 2) reveals that cluster I separates into 3 sub-states having unique structural and energetic properties. The largest sub-state in Level 2 comprises more than 6,000 conformations, and the internal energy distribution in this cluster is quite diverse (Figure 5; Level 2). Hence we use QAA to descend one more level in the conformational landscape. At Level 3 and Level 4 of QAA, we observe that the landscape splits into three and two sub-states respectively. The hierarchy in the energy landscape as revealed by QAA indicates that one can segment the highly complex conformational landscape of ubiquitin into energetically homogenous conformational sub-states. This successive homogenization in positional and energetic terms also provides for an intuitive understanding of the motions involved in ubiquitin binding, as illustrated above each panel in Figure 5. At Level 1, the fluctuations are global involving the pincer regions: 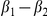 (red), 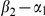 (cyan; R1), C-terminal tip of 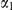 (R2; orange) and 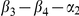 (blue) regions. At Level 2 the motions become localized to the protein binding loops: R1 albeit with lower amplitudes (see Movie S1 depicting the ubiquitin motions between the conformational sub-states). At Level 3 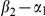 is coupled to R1 and at Level 4, R2 is coupled to R1.

The separation between the high- and low-energy conformations from each cluster, as identified by QAA, provides a unique opportunity to examine the biophysical relevance of the relative populations and its impact on ubiquitin binding. Note that at any given level of the conformational hierarchy, the presence of a minor population of conformations sharing either high- or low- internal energy. These minor populations deviate from the largest heterogenous cluster in exhibiting motions along functionally relevant regions. As one descends the conformational hierarchy, it becomes clear that the flexible regions of the protein do not change; only the amplitude of the actual conformational change changes (with proportional change in internal energy of the conformer). These changes in both motions and energetics allow ubiquitin to sample conformations that may in fact exceed the observed diversity in all of its bound conformations. Observe that the top 3 anharmonic modes of motion covers all of the conformational heterogeneity exhibited by the bound X-ray ensemble (Figure 4; blue diamonds). The hierarchy of motions in ubiquitin allow the protein to sample conformations that involve modulating the pincer regions (R1 and R2) to varying degrees. This subtle interplay between global conformational fluctuations (Level 1 motions) as well as its ability to modulate local motions (Levels 2 through 4) can thus enhance ubiquitin's ability to target multiple substrates [11].

Overall, QAA allows the identification of energetically homogenous sub-states as well as a multi-level hierarchy of internal motions for ubiquitin. In addition, conformational transitions identify how the binding regions are modulated between different sub-states in the hierarchy. These motions are directly relevant in the context of ubiquitin's ability to recognize multiple binding partners. In the next section, we will examine the ability of QAA to extract low dimensional representations of the conformational landscape and describe it in terms of a biophysically relevant order parameter.

### QAA reveals modulation of substrate-binding pocket in T4 lysozyme

T4 lysozyme catalyzes the hydrolysis of glycosidic bonds in polysaccharides from bacterial cell walls [46]. Lysozyme (164 residues) is composed of two individual sub-domains: N- and C-terminal linked by a single long 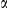-helical chain. The relative placement of the N- and C-terminal sub-domains forms a deep pocket where the ligand can bind. Ligand binding and release are associated with motions involved in opening and closing of this binding pocket relative to the N- and C-terminal sub-domains as evidenced from experiments and computational studies [46]–[48]. From a 120 ns simulation of lysozyme (Materials and Methods section) a total of 12,000 equally spaced conformations were analyzed using QAA. The C-terminal end residues 163–164) were excluded for QAA since these residues undergo large fluctuations. The original 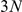 dimensional space for the C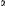 atoms was 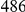 (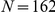); this was first projected onto a 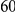 dimensional space using QHA (covering 70% of the overall variance) and then QAA was performed.

In addition to obtaining insights into the conformational sub-states in lysozyme, the motivation for this simulation was to test QAA on variety of criteria. First, it will help validate if QAA is robust to different implementations of force-fields (OPLS-AA [49], [50] force-field was used for lysozyme simulation, while AMBER *parm98* [51], [52] was used for ubiquitin and cyclophilin A simulations). Second, it will also illustrate if the sub-states identified using QAA can be mapped onto a physically observable order parameter, which is important when using low-dimensional representations. Given the relatively large binding pocket and well-documented motions, lysozyme provides an opportunity to evaluate if QAA can be used to isolate and characterize the sub-states involved in controlling the binding pocket. Finally, the time-scale of the lysozyme simulations allows the comparison of QAA (and its representation) to other techniques (see Discussion section).

**Figure 6:**
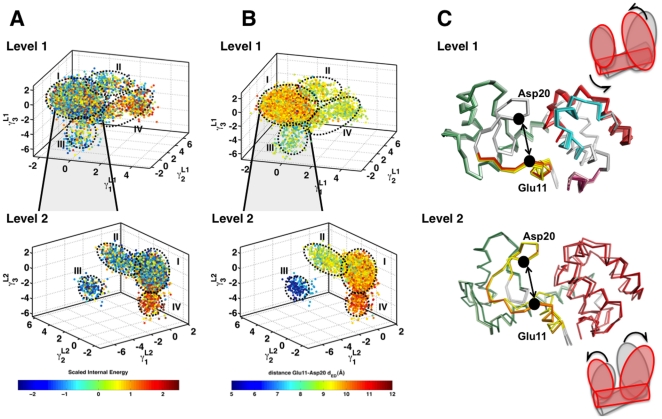
QAA reveals a hierarchy of sub-states in T4 lysozyme sharing similar internal energy and order parameter distributions. (A) The conformational hierarchy of lysozyme as described by QAA. Conformations are first projected onto the top three anharmonic modes (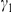, 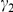 and 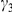) for each level of the hierarchy. Only two levels of the hierarchy are shown. Each conformer is painted with the scaled internal energy [44] described in the text. (B) To validate QAA can extract suitable order parameters, we painted each level of the hierarchy with an order parameter 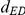 defined as the distance between the C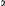 distance between catalytically important residues: Glu11 and Asp20. As illustrated, each conformational sub-state shares a remarkable similarity in the defined order parameters. In Level 1, sub-states II, III and IV share relatively smaller distance in between the catalytic sites; in Level 2, there is a clear separation in between the catalytic sites. (C) Beside each level, the motions involved in the first (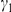) anharmonic mode is shown in a movie-like fashion. The frames of the movie (see SI movies) are colored according to the internal energy of the protein; darker shades represent higher-energy conformers. In Level 1, as shown in the cartoon-like representation at the top, we observe large-scale fluctuations in the larger lobe of the protein and the helix (shown in green), where as in Level 2, the motions are along both the lobes of the protein.

Similar to ubiquitin, the hierarchy reveals conformational wells that are homogenous in their internal energy distributions. As shown in Figure 6, Level 1 consists of four distinct conformational sub-states when organized along the top three anharmonic modes (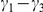). The largest cluster consists of over 80% of the conformers and the rest occupy the three smaller peripheral regions emerging from this cluster. An examination of these sub-states reveal that while clusters I, II and III consist of heterogenous energy distributions, cluster IV is enriched for higher energy conformers. Given the heterogenous population of conformers in Level 1, QAA was applied to this conformational well. In Level 2 of the hierarchy, the energy separation between the conformational sub-states become even more apparent: 73% of the conformers populate cluster I; others populate two low energy (clusters II and III) and one high energy (cluster IV). This homogeneity observed across the sub-states suggests that irrespective of the force-field (and the MD simulation package) used, QAA reveals intrinsic properties of the conformational landscape associated with both the internal dynamics and energetics of lysozyme.

The transitions described by QAA are also directly related to the relative motions between N- and C-terminal domains of lysozyme. To quantify these motions, we used an order parameter 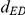 defined as the distance between the C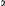 atoms of catalytic residues Glu11 and Asp20. Both residues are implicated in the catalytic mechanism; Glu11 protonates the glycosidic oxygen atom while Asp20 is crucial for stabilizing the reaction intermediate. Note that 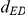 qualifies as a direct geometric observable from the simulation that quantifies the opening/closing of the binding cleft [33]. The conformers projected onto the top three anharmonic modes and colored by 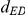 show clear separation across the sub-states. The homogeneity in the internal energy distributions and the 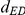 values are quantified further in Figure S4 and Figure S5. This homogeneity implies that for the set of chosen QAA basis vectors, the projections of the conformers clearly distinguish the increase/decrease in 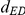 as the simulation progresses. Thus, a small number of QAA basis vectors can be used reliably to extract biophysically relevant order parameters from MD simulations.

The anharmonic modes of motion allow for a natural decomposition of the landscape that are directly coupled to the motions in the lysozyme binding pocket. While sub-states II, III and IV at Level 1clearly show a low 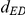, the largest conformational well consists of a heterogenous distribution of 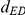, implying that the motions in Level 1 of the hierarchy identifies transitions associated with the decrease of 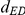. Moving along any QAA basis vector in this reduced dimensional space would entail a global breathing motion in lysozyme that brings both the N- and C-terminal sub-domains close to each other (Movie S2). In Level 2 of the hierarchy (based on iteratively applying QAA to cluster I from Level 1), there are more subtle changes in the protein's conformation that lead it to sample two conformational sub-states (II and IV in Figure 6). The motion along 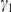 in Level 2 decreases 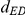 with motions detected along the C-terminal end of the protein, where as motion along 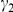 in Level 2 increases 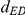, with motions. Thus, QAA can evaluate the suitability of an order parameter for obtaining biophysical insights and it can also distinguish how global and local motions may modulate different regions to achieve a functionally relevant conformation.

QAA provides detailed insights into how changes in 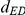 are directly related to the internal energetics of lysozyme. In Level 1 of the hierarchy, a global motion involving the entire protein leads to a higher energy state with a corresponding decrease in 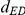. Although, only 4% of conformers sample this higher-energy state, the motions indicate the ability of lysozyme to sample this biologically relevant states even at equilibrium. In Level 2 of the landscape, we find that other collective fluctuations, more local than the ones described in Level 1, predominantly visible along the C-terminal sub-domain of lysozyme play a role in controlling the binding cleft conformation. Taken together, the motions indicate that both local and global motions are exquisitely coupled and activation of a particular mode can substantially alter lysozyme's energy landscape. The higher-energy conformers represent rare but conformationally accessible *excited* sub-states which are both relevant to the change in the binding cleft conformation. The rarity of these transitions is mainly associated with the overall internal stress in lysozyme resulting from the twisting motions in the N-terminal end and torsional motions in the C-terminal sub-domain. Thus, QAA enables the identification of biologically relevant rare-conformational transitions in the landscape. Although analysis of the variance using PCA based techniques also reveals similar motions (see Discussion section), QAA modes have provided an intuitive interpretation of motions that activate transitions from low to high energy sub-state (and vice-versa).

For lysozyme, QAA yields distinct energetically homogenous sub-states as well as separation between sub-states in terms of order parameters (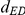). Note that the use of order parameter 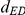 provides the utility of QAA as a general tool to distinguish various sub-states based on other parameters beyond internal energy (as demonstrated for ubiquitin). Similar to the observations from ubiquitin, the lysozyme landscape is also composed of sub-states that share common structural features which have direct relevance in binding to its substrate.

### Conformation sub-states explored during enzyme catalysis by cyclophilin A

Enzyme cyclophilin A is a peptidyl-prolyly isomerase (PPIase) as it catalyzes *cis/trans* isomerization of peptide bonds in small peptides and proteins [2], [26]. The enzyme's active-site, located on one face of the molecule, is formed by a pocket of hydrophobic residues including the conserved Phe113 and Ala101. This hydrophobic pocket allows the substrate proline residue to be held during the rotation of the amide oxygen preeceding the target proline residue, while hydrophilic residue Arg55 makes hydrogen bonds with the substrate [53]. The reaction mechanism of cyclophilin A has been the subject of experimental and computational studies as a prototypical system for investigating the interconnection between intrinsic dynamics and the enzyme mechanism [3], [14], [18], [19], [26]. NMR studies have indicated the rate of conformational fluctuations of the protein backbone, in several surface loop regions, coincidence with the substrate turnover step [14], [26]. Computational investigations have revealed the existence of a network of vibrations, formed by conserved residues, that connects the thermodynamical fluctuations of the surrounding solvent with the active-site [3], [19]. More recently, in a fascinating study hidden alternative conformations of cyclophilin A have been discovered that provide valuable insights into the promoting role of conformational fluctuations in the reaction mechanism of this enzyme [20].

**Figure 7:**
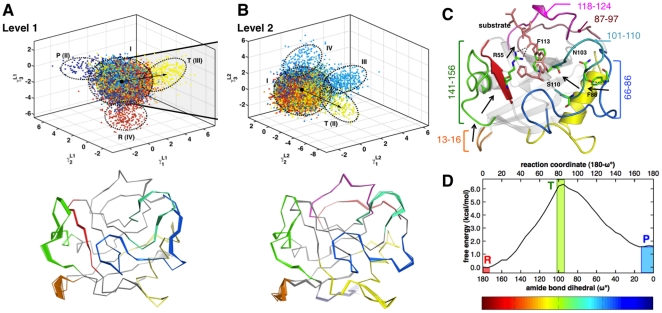
QAA describes conformational sub-states leading to transition state during catalysis in cyclophilin A. (A) Level 1 (top panel) of the catalytic landscape of cyclophilin A showing regions of high flexibility (bottom panel). Each conformation from the simulation is painted with the reaction coordinate (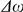). Note the separation between the ground-state conformers (cluster II) and transition state conformers (cluster III). Observe that flexible loops 12–15, 26–40, 54–60, 66–76 and 101–110 show relatively large motions leading to the transition state. Most of these regions have also been previously implicated in enabling catalysis by allowing the enzyme and substrate peptide to interact favorably so that the isomerization can proceed further. (B) In Level 2 (top panel), motions leading to the transition state activate complementary regions in addition to motions observed in Level 1. Note that the motions in the flexible loops highlighted in Level 1 undergo lower amplitude motions; however, flexible loops 77–96, 120–126 show pronounced fluctuations at this level. Note that in both (A) and (B), the color scale from the amide bond dihedral is used to paint the conformers; the transition state conformers are painted in light green. (C) The coupling observed confirms previous studies which identifies a network of coupled motions extending from the flexible surface regions all the way to the active site connected by hydrogen bonds. Note the motions of Phe83 and Asn103 are critical for enzyme function. (D) shows the free-energy profile for the *cis/trans* isomerization of the bound peptide.

QAA allows the identification and characterization of the conformational sub-states associated with the *cis/trans* isomerization catalyzed by cyclophilin A (Figure 7). As previously described the reaction pathway was modeled by using the amide bond dihedral angle (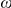) as reaction coordinate [18], [19]. The change from the reactant state (*trans*, 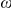 = 180°) to the product state (*cis*, 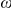 = 0°) was modeled by using a series of umbrella sampling runs with 37 independent runs. 18,500 enzyme conformations (with bound substrate) collected during these runs and were first projected on to the top 60 QHA dimensions (covering 70% of the overall variance); and then analyzed using QAA. Note, this methodology provides exploration of a non-equilibrium process as compared to the equilibrium state that is explored in free MD simulations of ubiquitin and lysozyme. Additionally, cyclophilin A system consisted of the protein enzyme bound to the catalyzed substrate.

The multi-level hierarchy of the protein conformations along the reaction pathway of cyclophilin A also indicated the presence of the conformational sub-states, as seen in both ubiquitin and T4 lysozyme. As depicted in the Figure 7A, at Level 1 the majority of the conformations fall in a central cluster but there are 3 additional clusters that are observed. Note that the scheme for painting here is different than the other two systems; here the conformations are colored based on the value of the reaction coordinate the cyclophilin A explores. This coloring scheme provide a more meaningful interpretation as it corresponds to the movement of enzyme over the reaction pathway (coordinate). A careful characterization indicates the enzyme intrinsic ability is to explore conformation that correspond to various sections of the reaction pathway, in addition to separate (and intuitively) the lower energy states corresponding to the reactant and product states. Note, these clusters correspond to the lower energy states in the free energy profile for the *cis/trans* isomerization reaction.

The most interesting feature revealed by QAA is the presence of a separate conformational sub-state that shows a significant presence of the structures that correspond to the transition state during the enzyme reaction. This region is colored light green in the figure, and note that as previously indicated the transition state for this reaction corresponds to 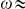 90°–100°) (Figure 7D), and the top of the free energy profile [18], [19]. At Level 2 (Figure 7B), a further decomposition of the largest cluster at Level 1 also indicates the presence of additional sub-states with a large sub-state corresponding to the enzyme conformations with features that correspond to the transition state. Both at Levels 1 and 2, the existence of separate sub-states with conformations that correspond to this region of the reaction pathway that correspond to the transition state provides vital insights into the conformational landscape of this enzyme. The movement along the vectors connecting the clusters (indicated by arrows in the figure), correspond to internal protein motions that allow the enzyme to sample conformations that have feature suitable to promote the transition state [14], [26]. This is consistent with the recent observation of the hidden alternate conformations that are explored by the enzyme during the catalytic mechanism [20]. Note, that even though naturally these motions are sampled by cyclophilin A at a much slower rate (hundreds of microseconds, corresponding to the time-scale of the reaction), the use of a reaction coordinate with umbrella sampling allows the enzyme to sample these higher energy states more frequently in our simulations.

The comparison of enzyme conformations between these clusters (both at Level 1 and 2) provide insights into the intrinsic dynamical features of the enzyme. The movement along the vectors between these clusters (corresponding to rare-conformational transitions or slow conformational fluctuations) show that the largest motions is located in the protein regions that are colored in the Figure 7B. These include the cyclophilin A regions 13–16, 55–60, 66–86, 87–97, 101–108, and 141–156, which have been previously implicated in a network of coupled protein vibrations. This observation is consistent with the previous observations from the computational (based on QHA) and NMR studies [14], [19]. Previously, it was proposed that these highly flexible regions are connected by a network of conserved network residues that originate on the surface regions and reach all the way into the active-site. Particularly, the surface residue Phe83 (located in the flexible region 66–86) is connected to Asn103 by a conserved network hydrogen bond. Additional interactions (indicated by black arrows) relay the motions into the active-site, where they mediate the enzyme-substrate interactions through residues such as Phe113. Movies describing these motions are depicted in Movie S3.

A careful analysis at Level 2 also indicates that the conserved active-site Phe113 switches conformation from one cluster to another cluster. This induces an important change in the hydrophobic environment in the active-site. Similarly on the other side, the region 13–16 is interconnected to 141–156 and 55–60 eventually allowing catalytically important Arg55 to mediate the substrate orientation through two important hydrogen-bonds (Figure 7C). As previously observed small changes in the active-site environment have important implications for the reaction mechanism [53]. Overall, QAA allows the exploration of cyclophilin A conformational landscape associated with the *cis/trans* isomerization reaction. The decomposition of the landscape in sub-states allows identification of the conformations that have features relevant to the transition state, and therefore, allows identification of the subtle changes in various dynamically relevant residues. Ongoing analysis of reactive trajectories as they visit these sub-states will allow us to quantify the rates of interconversion and its connection to the reaction kinetics.

### Intuition for energetic homogeneity in sub-states described by QAA

Based on the results from three different proteins, we have illustrated the ability of QAA to delineate events linked to molecular recognition of binding partners and enzyme catalysis under equilibrium and non-equilibrium conditions respectively. In each case, QAA identified energetically coherent conformational sub-states and functionally relevant global motions.

The energetic homogeneity in the sub-states discovered by QAA is a consequence of pursuing super- and sub-Gaussian fluctuations explicitly. Gaussian fluctuations arise when atoms are moving under the influence of an harmonic potential well, whereas super- and sub-Gaussian fluctuations are sampled from wells that could have non-harmonic shapes including square well, double-well/multi-well. This is consistent with previous studies that evaluated the nature of atomic fluctuations from picosecond time-scale MD simulations [54]–[56]. Further, in the case of two dimensional data shown in Figure 3 (as well as Figure S2), rare fluctuations represent a separation in the energetic properties (high to low or vice-versa). QAA in its pursuit of higher-order statistics can, therefore, distinguish these different shaped potentials and thus, provide a natural means of decomposing the complex energy landscape into energetically homogenous sub-states. The identification of rare-conformational transitions as well as collectively fluctuating regions in the protein is of functional importance. Rare-conformational transitions between sub-states have biophysical relevance in both binding and catalysis, as we have demonstrated in this paper for ubiquitin and cyclophilin A respectively. Further, NMR and more recently X-ray crystallography have at various levels implicated the presence of small populations of such rare conformational changes as being important for its function in several proteins [9], [20].

### Coupling between QAA modes

**Figure 8:**
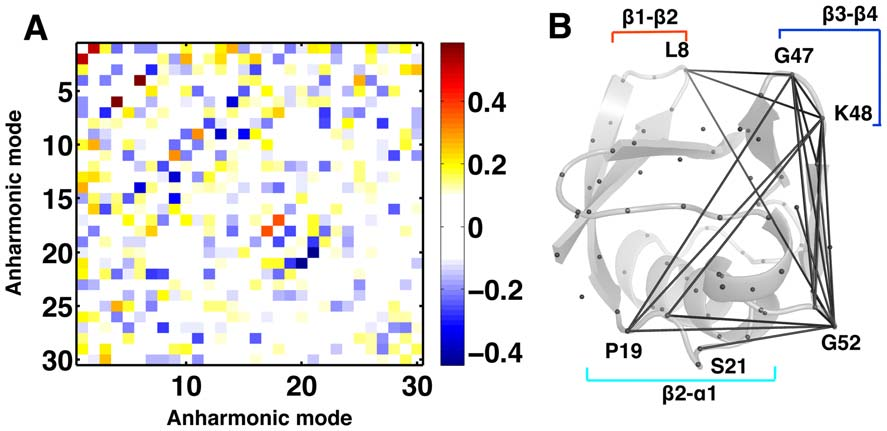
Coupling between anharmonic (QAA) modes of motion. (A) Most anharmonic modes are weakly coupled as indicated by the coupling co-efficients (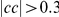). (B) An example of anharmonic coupling between modes 1 and 2 (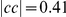) for ubiquitin shows spatially coupled regions in the protein. Observe the long-range coupling between R1 and 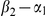. C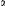 atoms are shown as gray spheres and residues commonly activated by modes 1 and 2 are marked and connected by gray lines.

Unlike QHA, the anharmonic modes from QAA need not be orthogonal. Hence, it is possible for these anharmonic modes to activate each other depending on their intrinsic coupling. The coupling coefficient or the interaction strength can be measured as 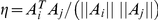. As depicted in Figure 8 for ubiquitin most modes are weakly coupled [57]. For example, consider QAA modes 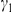 and 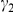 at Level 1 in ubiquitin: 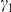 shows global fluctuations involving regions R1 and R2 whereas 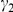 activates motions along 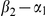 and R1. As illustrated in Figure 8 (right), commonly activated residues and their interactions were identified by thresholding the matrix 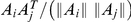 based on their interaction strength. These specific activation patterns along particular anharmonic modes of motion may provide insights into how energy transfers from local to global conformational fluctuations [58]. One way to test the coupling empirically is to use biased MD simulations where energy is pumped into a specific QAA mode and observe how it propagates into the other coupled modes [32].

QAA has clear advantages over established methods in segmenting a protein's energy landscape into multi-scale, energetically coherent conformational sub-states, and in identifying novel reaction coordinates. It is also important to note that choice of non-orthogonality amongst the basis vectors in QAA does not limit its ability to define suitable order parameters. Indeed, as we have shown, the 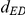 parameter is separated well within the conformational sub-states for lysozyme (see Figure 6). Further, in the case of cylcophilin A, the conformations clearly identify a separation between the ground and transition states based on the reaction coordinate (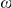; Figure 7). Thus, in terms of discovering relevant order parameters, the use of QAA provides not only biophysical rigor but also enhances the interpretability of the potential energy landscape. It remains to be seen whether the defined order parameters can be reliably used for umbrella sampling approaches, which will be studied in the near future.

### Comparison of QAA to other methods

With QAA we emphasized two statistical properties of internal protein motions: anharmonicity and non-orthogonality. Previous work characterizing anharmonicity in MD simulations used picosecond length trajectories [54], [56]. Anharmonic statistics were also used to refine X-ray crystallographic data [59]. In comparison, our work uses long, extensive atomistic level MD simulations of length up to 0.5 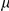
s as well as a reaction pathway sampling method that allows conformational sampling for an enzyme reaction at 0.1 milliseconds.

For investigating protein dynamics in collective coordinate space, a number of techniques have been developed for identifying biophysically meaningful directions of the conformational landscape using orthogonal motion basis [60]. An obvious approach is is to approximate the conformational landscape as a single harmonic well with known second derivatives of the potential function, as in normal mode analysis (NMA) [61]–[63]. A closely related approach is to resolve the second-order statistics of the collective coordinates with approaches based on principal component analysis (PCA) [64], such as QHA and essential dynamics [65]. NMA- and PCA-based approaches are popular due to their inherent simplicity: beginning with a single X-ray crystal structure, an experimental ensemble of structures, or MD simulation trajectory, it is possible to obtain useful insights into the internal motions and intrinsic flexibility of a protein [11], [12]. While useful, the general suitability of these methods for interpreting anharmonic motions or reliably isolating conformational sub-states has been questioned [57], [66]–[68].

In the results section, we have motivated how QAA differs from QHA in terms of interpreting overall motions using the joint distributions of positional fluctuations in two dimensions. In higher dimensions (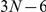; 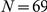) for ubiquitin, QHA describes the overall fluctuations involving global motions in the primary binding regions (R1 and R2). However, when we paint the internal energy for each conformation projected onto the top three harmonic modes of motion, we observe that energetic homogeneity is lacking between the conformations (Figure S6 and see description in Text S2). Thus, from the perspective of overall motions, even though QHA implicates the flexible regions of the protein, it cannot accurately single out conformational transitions between energetically homogenous sub-states. In QHA, this is a consequence of blind pursuit of variance and imposition of an orthonormal basis representation. QAA, by using higher-order statistics can easily separate if the atomic fluctuations are sub- or super- or purely Gaussian. Further, by not imposing an orthonormal basis representation, QAA can pursue directions in the complex multi-dimensional space that are clearly relevant to the protein's function. While this observation is true for a cartesian coordinate representation of the protein conformation, we expect it to also hold in an internal coordinate based representation. Interestingly, a comparison between QAA and dihedral PCA shows that although dihedral PCA separates the conformational space better than PCA based approaches, it still does not provide insights into energetically homogenous sub-states (for a comparison of QAA with dihedral PCA [69]–[71] see Figure S7 and the corresponding description Text S2). Further, it is not only the top 3 anharmonic modes of QAA (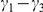) that identify energetically homogenous sub-states, but lower amplitude modes also identify directions in the landscape that lead to energetically homogenous sub-states (Figures S3 and S4).

The existence of nonlinearly related motions has already motivated mutual information (MI) based decoupling approach called full correlations analysis (FCA) for detecting higher-order correlations [34] which is in turn based on independent component analysis [38], [72], [73] a popular approach in signal processing and other non-linear methods [74]–[77]. To avoid costly entropy calculations required by FCA, the work here pursues kurtosis, a statistic which approximates mutual information. Note that for lysozyme (Figures S8), a comparison between negentropy and kurtosis reveals almost similar distributions, indicating that the information contained by both techniques are indeed similar. It must also be pointed out that both FCA and QAA start out by projecting the conformational landscape into a reduced dimension representation using PCA. In addition, both methods retain explicit emphasis on anharmonicity. However, unlike FCA, QAA permits non-orthogonal motion representation. For joint distributions in positional deviations, FCA does not recover the intrinsic orientation of the dependencies observed because of orthogonal choice in representing motions (see Figure S2 and description in Text S1). Further, the orthogonal choice need not provide the clear separation in terms of order parameters as shown in Figure S9 and Text S3.

Overall, by pursuing higher-order statistics and anharmonicity of protein motions, it has been possible to obtain novel insights into the conformational sub-states and transitions between these sub-states that would have been otherwise difficult (using second-order correlation techniques such as QHA and dihedral PCA). Further, examining the non-orthogonal dependencies in atomic fluctuations delineates energetic differences within and between various sub-states in the landscape (Figure 3 and Figure S2). The non-orthogonal directions also enable identification of coupling between different regions of the protein and inter-dependencies between different protein motions.

## Discussion

Proteins are not rigid structures but intrinsically capable of exploring an ensemble of conformations, enabled by a wide range of internal motions. The role of these conformational fluctuations, if any, in the designated functions of the proteins including biomolecular recognition and enzyme catalysis has been challenging to characterize. The challenge partly arises from the fact that the internal protein motions occur on a wide range of time-scales, while the individual experimental instruments only provide access to information corresponding to narrow windows of resolution. Computational methodology recently provided vital insights, due to its ability to provide atomistic level information on a wide range of time-scales. Emerging evidence has indicated the possibility that certain parts of the conformational ensembles (or sub-states) may posses structural features that could be relevant and even vital for the mechanism of designated function. Unfortunately, due to the low probability of finding these conformations in the multi-level hierarchy of a protein's conformational landscape, makes the identification and characterization of these sub-states rather difficult.

In this paper, a new methodology QAA is described that allows automated discovery of a hierarchy of sub-states associated with the conformational ensemble of proteins. Utilizing atomistic level MD simulations of proteins or protein in association with other molecules (such as binding partners or enzyme-substrate complex) as input, this methodology pays close attention to the anharmonic nature of internal protein motions and pursues the higher-order statistics of the internal motions. One of the most important advantages of this approach is that it allows clean separation between the conformational sub-states, by projecting the conformations sampled during the MD simulations in a lower dimensional space represented by QAA vectors. Characterization of the populations in these sub-states for any relevant properties (such as internal energy, distance order parameter, or reaction coordinate) allows the detailed characterization. In addition, to identifying these sub-states, the motions associated within the sub-states and inter-conversion between the sub-states provide new insights in to the inter-relationship between protein structure, motions and function.

The use of QAA shows the equilibrium motions of human ubiquitin at the 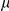
s-scale exhibit significant higher-order correlations both for individual atoms and collective fluctuations in the protein. The identified conformational sub-state decomposition revealed a natural hierarchy of fluctuations that are important for ubiquitin to bind diverse substrates. By characterizing the anharmonic fluctuations, QAA revealed the presence of conformational sub-states with different internal energies that are homogeneous within and heterogenous between sub-states. The unique structural features identified by QAA elucidate the mechanism of binding motions in ubiquitin. For lysozyme, QAA was also able to identify sub-states that not only were energetically distinct, but analysis based on a relevant order parameter was able to describe motions that were directly tied to the substrate-binding pocket.

For reactive systems such as the enzyme cyclophilin A, QAA allows characterization of conformational sub-states along the reaction pathway. A hierarchical description of the sub-states along the reaction pathway identifies sub-states with structural and dynamical features critical for attainment of the transition state. Inspection of conformational transitions that allow the enzyme to move from one sub-state to another represents rare-conformational transitions that are intrinsic properties of cyclophilin A. In each of these functionally relevant transitions provides further biophysical insights into the previously identified network of coupled vibrations [18]. In addition, the mapping of localized motions to the global fluctuations QAA provides insights into how each protein has effectively been designed to achieve their target function by utilizing those motions that allow the protein to explore energetically coherent sub-states. It will be of interest to analyze the energetic coupling between anharmonic modes as well as free-energy changes required for such conformational diversity and transitions between sub-states.

## Materials and Methods

### Ubiquitin Simulations


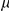
s timescale simulations for ubiquitin were carried out as described in previous work [22]. Ubiquitin simulations were performed using AMBER molecular mechanics package and the *parm98* [51], [52] force-field in explicit solvent based on SPC/E water model [78], [79]. Note the suitability of the *parm98* force-field for investigating protein dynamics has been verified previosuly [18]. Starting with eight different crystal structures [PDB codes: 1UBQ; 1P3Q (chain U); 1S1Q (chain B); 1TBE (chain B); 1YIW (chain A); 2D3G (chain B); 2FCQ (chain B); and 2G45 (chain B)] that covered the structural diversity of ubiquitin's conformation, stable MD trajectories were generated. Each simulations was run for 62.5 ns, collectively accounting for 0.5 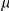
s sampling. This approach of using short MD trajectories to obtain information about longer time-scales was used by Caves and co-workers [80], which showed that time-scale accessible to MD simulations from a single 1 ns run was shorter than the time-scale accessible to a collection of 10 individual MD runs that lasted 100 ps. Further, Shirts and Pande [81] also showed that using a large number of smaller MD runs could approximate long time-scale fluctuations derived from a single long MD run.

### Lysozyme Simulations

MD simulation for T4 lysozyme were initiated from the crystal structure 2LZM [46]. For this simulation, we used the recently developed Desmond [82] package and OPLS-AA force-field [49], [50]. After determining the protonation state for each residue at pH 7.0, hydrogens were added to the protein using Maestro software. After neutralizing the charge of the system using eight Cl^−^ ions, the protein was immersed in a pre-equilibrated SPC [78], [79] water box such that the distance between the box-boundary and the surface of the protein was at least 10 Å. The system was then subjected to a series of short MD simulations to allow it to equilibrate at 300 K. First, the solute was held fixed and the solvent was energy minimized using conjugate gradient technique for about 500 steps. The solute was energy minimized to release any conflicting contacts using a similar procedure. A small MD simulation under constant pressure (for 20 ps) with gradual increase in temperature to 300 K was then performed with the solvent molecules being unrestrained. This was followed by two additional rounds of constant volume equilibration simulations to allow the system to reach a stable conformation at 300 K. A final MD run of about 200 ps was then performed under constant volume conditions to ensure that the system was stable.

All production runs were performed using NVE conditions with periodic boundary conditions. Bond lengths to hydrogens were maintained through out the simulations with SHAKE algorithm. Electrostatic interactions were evaluated using Particle Mesh Ewald (PME) method and the long-range interactions were truncated at 10 Å. A single continuous MD production run of lysozyme was carried out for a total of 120 ns with snapshots being saved every 10 ps, resulting in a total of 12,000 snapshots.

### Cyclophilin A

The human cyclophilin A was modeled as previously described with peptide substrate *His*–*Ala*–*Gly*–*Pro*–*Ile*–*Ala* based on the PDB structure 1AWQ [18]. The reaction pathway was modeled based on amide bond dihedral angle (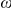) as reaction coordinate (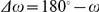); 37 windows (in 5° decrements) were used to map the reaction from the reactant state (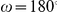) the product state (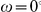). Each window was simulated for 200 ps and 500 structures from each MD simulation were collected. Therefore, a total of 18,500 conformations were used for QAA. See reference [18] for complete simulation details.

## Supporting Information

Figure S1
**Long-tail distributions at shorter time-scales; side-chains have greater anharmonicity than backbone atoms.** Anharmonic distribution of positional deviations (Å) from ubiquitin MD simulations at 5 ns and 50 ns. For each atom, the positional displacement from the time-averaged position was calculated at 50 ps intervals. The same bin size (0.54 Å) was used for all histograms. Distributions correspond to: C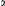 (red), Gaussian fit to C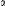 (dotted red), side-chains (light blue) and all-atoms (black). The probability distributions of positional deviations [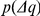] are plotted in log-scale.(TIF)

Figure S2
**QAA captures intrinsic non-orthogonal directions pointing towards energetically coherent directions in the landscape; QHA and FCA do not.** For the ubiquitin simulation (0.5 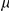
s), (A) residues 2 and 14 exhibit Gaussian-like fluctuations in the 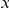 and 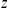 directions respectively. When pairwise distributions are Gaussian-like, QHA (black) and FCA (purple) basis vectors [34] align well with the intrinsic orientation of the data. Residues 31 and 45 are anharmonic in the (A) 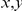 and (B) 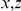 directions, illustrative of modeling challenges for intrinsically non-orthogonal data. QHA (black) and FCA (purple) cannot accurately describe these orientations, whereas QAA (red arrows) align well with the non-orthogonal directions and point towards homogenous energy distributions. (D–F) Distributions identical to (A–C) are colored according to scaled interaction energies (as explained in the main text). QAA basis vectors align with energetically coherent sub-states. In (A–F), dotted lines indicate contours of the non-Gaussian directions in positional fluctuations. Energy distributions are also shown below associated joint distributions; in each the color range is thresholded above and below 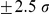 for visual clarity. All spatial units are in Å. For each residue pair a total of 100,000 conformers were used from the 0.5
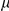
s simulations.(TIF)

Figure S3
**Projections of ubiquitin simulation (0.5** 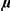 **s; 10,000 conformations) onto eight top quasi-anharmonic modes (** 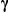 **) from QAA illustrate distinct separation in energy distributions.** Structures are colored according to scaled (zero mean, unit variance) non-bonded energies, that is, the sum of electrostatic and van der Waals energy terms. Color bins are thresholded at 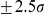 (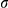 - standard deviation). Ellipses indicate clusters determined by mixture of Gaussian (MoG) model [41]. Each cluster is indicated by a colored ellipse whose major and minor axes correspond respectively to the first two principal components of each cluster. Neighboring panels show histograms of energy values within each cluster. Note the colors of the ellipse and histogram match. For each projection, the largest and most energetically heterogenous cluster (brick ellipse) is not included in the histogram to clarify energetic coherency of the remaining (less populated) conformational sub-states. Boxes above the histograms show both the means (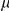) and standard deviations (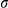) of energy distributions in respective clusters.(TIF)

Figure S4
**Lysozyme simulation projected onto eight top quasi-anharmonic modes (** 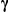 **) from QAA illustrate distinct separation in energy distributions.** Structures are colored according to scaled internal energies, as explained in the main text. Color bins are thresholded at 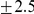 standard deviations. Ellipses indicate clusters determined by mixture of Gaussian (MoG) model [41]. Each cluster is indicated by a colored ellipse whose major and minor axes correspond respectively to the first two principal components of each cluster. Neighboring panels show histograms of energy values within each cluster. Note the colors of the ellipse and histogram match. For each projection, the largest and most energetically heterogenous cluster (brick ellipse) is not included in the histogram to clarify energetic coherency of the remaining (less populated) conformational sub-states. Boxes above the histograms display means (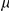) and standard deviations (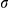) of energy distributions in respective clusters. QAA commonly resolves and separates high and low energy sub-states. Projection systems 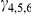 and 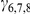 show clusters (blue ellipses) with mean energies far from global energetic mean (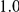 and 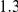 respectively versus 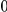), indicating the QAA modes' ability to characterize internal energetics. Compare with Figure 16, where highest resolved cluster mean energy is 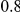 (FCA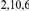).(TIF)

Figure S5
**Lysozyme simulation projected onto six QAA coordinate systems.** Axis labels correspond to mode indices ranked by fluctuation magnitude, and were chosen sequentially. Structures are colored according to d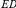, the distance between catalytic sites Asp11 and Glu20. Ellipses indicate clusters determined by mixture of Gaussian (MoG) model [41]. Each cluster is indicated by a colored ellipse whose major and minor axes correspond respectively to the first two principal components of each cluster. Neighboring panels show histograms of distances (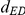) within each cluster. The colors of the ellipse and histogram match. Note the clear separation between the conformational clusters showing differences in distance (Asp11 to Glu20) distributions.(TIF)

Figure S6
**Lack of homogeneity in the internal energy distributions of QHA.** For the 0.5 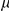
s simulations of ubiquitin (10,000 conformations), the top three basis vectors from QHA (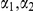 and 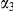) are depicted here. Projection of each conformation is colored by the scaled internal energy (as described in the main text). Note the apparent lack of clear separation between clusters when compared to QAA (main text, Figure 5).(TIF)

Figure S7
**Ubiquitin landscape represented by the first three basis vectors using dihedral PCA** [**69**] **from the 0.5** 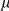 **s simulations (10,000 conformations).** Projected conformations show the presence of spatial clusters. However, when colored by the scaled internal energy, energetic homogeneity is lacking, unlike in the analogous QAA-based clusters (main text, Figure 5).(TIF)

Figure S8
**Lysozyme simulations projected onto FCA basis.** (**A**) We follow the protocol used in [34] to consider six projections from FCA (from Figure 12). Axis labels correspond to mode indices ranked by negentropy. Plots and clustering follow the protocol in Figure 12. Excepting FCA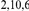 and FCA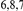, most projections poorly resolve energetic differences between clusters. (**B**) Comparison of FCA and negentropy for top 100 FCA modes. Circles indicate the modes selected for the projection coordinates in panel (A) and are sized according to the variance of the associated modes. Note that variance is not a reliable indicator of anharmonicity. (**C**) Correlation between negentropy and kurtosis for the top 100 FCA modes. Of these modes, 85 display Gaussian statistics (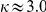 and negentropy 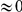, boxed in grey), suggesting that modes selected by either criteria (kurtosis or negentropy) signify key anharmonic directions.(TIF)

Figure S9
**Analysis of Lysozyme simulations using Full Correlation Analysis.** (**A**) Lysozyme simulation projected onto six full correlation analysis (FCA) coordinate systems (Fig. 12) according to procedure in [34]. Axis labels correspond to mode indices after ranking by negentropy. Conformations are colored by the distance between catalytic residues as shown in the previous plot. Observe that the separation between the clusters according to 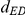 is not as clear as in Figure 13.(TIF)

Movies S1For ubiquitin, the movies depict the motions of C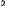 atoms for residues 2-70. Internal motions of ubiquitin are filtered along the 0.5 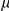
s MD simulation along the top-most anharmonic mode (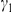) at each level (Level 1, Level 2, Level 3 and Level 4) of the hierarchy as illustrated in Figure 5 of the main text. Observe that motions become more local as one descends the hierarchy. The regions showing largest fluctuations are highlighted for visual clarity.(MPG)

Movies S2For T4 lysozyme the large-scale motions for Level 1 and Level 2 (shown in Figure 6 of the main text) are shown here. Note that the motions here depict movements of the substrate binding regions very clearly. Also note that the motions in Level 2 show a pronounced opening of the binding cleft, as indicated by an increase in the 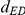 order parameter (described in the text). The movies also highlight the two sub-domains as well as the relevant motions between the sub-domains that cause the opening and closing of the substrate binding pocket.(MPG)

Movies S3 For the enzyme cyclophilin A, the movies depict movements of the highlighted regions in Figure 7. QAA modes chosen for our analysis at both Levels 1 and 2 are involved in transiting from the heterogenous conformational well (cluster I) to the transition state (cluster III) indicated by the arrow in Figure 7. The movies highlight key regions in cyclophilin A that are linked to the catalytic activity of the enzyme as observed from previous studies [18], [19]. For visual clarity the substrate is depicted in a stick representation to provide the viewer with a perspective of the catalytic site in cyclophilin A.
(MPG)

Text S1 Intuition for why QAA finds energetically coherent sub-states
(PDF)

Text S2 Comparison of QAA with dihedral PCA
(PDF)

Text S3 Comparing QAA with Full-Correlation Analysis
(PDF)

Text S4 Movies from QAA for Ubiquitin, Lysozyme and Cyclophilin A
(PDF)

## References

[1] Cannon WR, Benkovic SJ (1998). Solvation, reorganization energy, and biological catalysis.. J Biol Chem.

[2] Henzler-Wildman K, Kern D (2007). Dynamic personalities of proteins.. Nature.

[3] Agarwal PK (2006). Enzymes: An integrated view of structure, dynamics and function.. Microbial Cell Factories.

[4] Markwick PRL, Bouvignies G, Blackledge M (2007). Exploring multiple timescale motions in protein GB3 using accelerated molecular dynamics and NMR spctroscopy.. J Am Chem Soc.

[5] Elber R, Karplus M (1987). Multiple conformational states of proteins: A molecular dynamics analysis of myoglobin.. Science.

[6] Frauenfelder H, Parak F, Young RD (1988). Conformational sub-states in proteins.. Annu Rev Biophys Biophys Chem.

[7] Frauenfelder H, Sligar S, Wolynes P (1991). The energy landscapes and motions of proteins.. Science.

[8] Fenimore PW, Frauenfelder H, McMahon BH, Parak FG (2002). Slaving: Solvent fluctuations dominate protein dynamics and functions.. Proc Natl Acad Sci U S A.

[9] Boehr D, McElheny D, Dyson H, Wright P (2006). The dynamic energy landscape of dihydrofolate reductase catalysis.. Science.

[10] Benkovic SJ, Hammes GG, Hammes-Schiffer S (2008). Free-energy landscape of enzyme catalysis.. Biochemistry.

[11] Lange OF, Lakomek NA, Fares C, Schroder GF, Walter KFA (2008). Recognition dynamics up to microseconds revealed from an RDC-derived Ubiquitin ensemble in solution.. Science.

[12] Bahar I, Chennubhotla C, Tobi D (2007). Intrinsic dynamics of enzymes in the unbound state and relation to allosteric regulation.. Curr Opin Struct Biol.

[13] Benkovic SJ, Hammes-Schiffer S (2003). A perspective on enzyme catalysis.. Science.

[14] Eisenmesser EZ, Millet O, Labeikovsky W, Korzhnev D, Wolf-Watz M (2005). Intrinsic dynamics of an enzyme underlies catalysis.. Nature.

[15] Kamath G, Howell EE, Agarwal PK (2010). The tail wagging the dog: Insights into catalysis in R67 dihydrofolate reductase..

[16] Agarwal PK, Billeter SR, Rajagopalan PTR, Hammes-Schiffer S, Benkovic SJ (2002). Network of coupled promoting motions in enzyme catalysis.. Proc Natl Acad Sci USA.

[17] Bosco DA, Eisenmesser EZ, Pochapsky S, Sundquist WI, Kern D (2002). Catalysis of cis/trans isomerization in native HIV-1 capsid by human cyclophilin A.. Proc Natl Acad Sci U S A.

[18] Agarwal PK, Geist A, Gorin A (2004). Protein dynamics and enzymatic catalysis: Investigating the peptidyl-prolyl cis/trans isomerization activity of cyclophilin A.. Biochemistry.

[19] Agarwal P (2004). Cis/trans isomerization in HIV-1 capsid protein catalyzed by cyclophilin A: insights from computational and theoretical studies.. Proteins: Struct Func Bioinform.

[20] Fraser J, Clarkson M, Degnan S, Erion R, Kern D (2009). Hidden alternative structures of proline isomerase essential for catalysis.. Nature.

[21] Mchaourab HS, Oh KJ, Fang CJ, Hubbell WL (1997). Conformation of T4 lysozyme in solution. hinge-bending motion and the substrate-induced conformational transition studied by site-directed spin labeling.. Biochemistry.

[22] Ramanathan A, Agarwal PK (2009). Computational identification of slow conformational fluctuations in proteins.. J Phys Chem B.

[23] Petsko GA, Ringe D (2000). Observation of unstable species in enzyme-catalyzed transformations using protein crystallography.. Curr Opin Chem Biol.

[24] Boehr DD, Dyson HJ, Wright PE (2006). An NMR perspective on enzyme dynamics.. Chem Rev.

[25] Hammes GG (2002). Multiple conformational changes in enzyme catalysis.. Biochemistry.

[26] Eisenmesser EZ, Bosco DA, Akke M, Kern D (2002). Enzyme dynamics during catalysis.. Science.

[27] Faure P, Micu A, Perahia D, Doucet J, Smith JC (1994). Correlated intramolecular motions and diffuse x-ray scattering in lysozyme.. Nat Struct Mol Biol.

[28] Chen Y, Hu D, Vorpagel ER, Lu HP (2003). Probing single-molecule T4 lysozyme conformational dynamics by intramolecular fluorescence energy transfer.. J Phys Chem B.

[29] Schwartz SD, Schramm VL (2009). Enzymatic transition states and dynamic motion in barrier crossing.. Nat Chem Biol.

[30] Arora K, Brooks CL (2009). Functionally important conformations of the Met20 loop in dihydrofolate reductase are populated by rapid thermal fluctuations.. J Am Chem Soc.

[31] Garcia-Viloca M, Truhlar DG, Gao J (2003). Reaction-path energetics and kinetics of the hydride transfer reaction catalyzed by dihydrofolate reductaseâ€.. Biochemistry.

[32] Agarwal PK (2005). Role of protein dynamics in reaction rate enhancement by enzymes.. J Am Chem Soc.

[33] Hub JS, de Groot BL (2009). Detection of functional modes in protein dynamics.. PLoS Comput Biol.

[34] Lange O, Grübmuller H (2007). Full correlation analysis of conformational protein dynamics.. Proteins: Struct Func Bioinform.

[35] Hastie T, Tibshirani R, Friedman J (2009). The elements of statistical learning: Data mining, inference and prediction..

[36] Karplus M, Kushick JN (1981). Method for estimating the configurational entropy of macromolecules.. Macromolecules.

[37] Phillips J, Braun R, Wang W, Gumbart J, Tajkhorshid E (2005). Scalable molecular dynamics with NAMD.. J Comp Chem.

[38] Cardoso JF (1999). High-order contrasts for independent component analysis.. Neural Computation.

[39] Golub GH, Van Loan CF (1996). Matrix Computations..

[40] Hochstrasser M (1996). Ubiquitin-dependent protein degradation.. Annu Rev Genet.

[41] McLachlan G, Basford K (1988). Mixture Models: Inference and applications to clustering..

[42] Nabney IT (2003). NETLAB: Algorithms for Pattern Recognition..

[43] Bakan A, Bahar I (2009). The intrinsic dynamics of enzymes plays a dominant role in determining the structural changes induced upon inhibitor binding.. Proc Natl Acad Sci U S A.

[44] Kong Y, Karplus M (2007). The signaling pathway of rhodopsin.. Structure.

[45] Kong Y, Karplus M (2009). Signaling pathways of PDZ2 domain: a molecular dynamics interaction correlation analysis.. Proteins: Struct Func Bioinform.

[46] Weaver L, Matthews B (1987). Structure of bacteriophage T4 lysozyme refined at 1.7 Å resolution.. J Mol Biol.

[47] Post CB, Brooks BR, Karplus M, Dobson CM, Artymiuk PJ (1986). Molecular dynamics simulations of native and substrate-bound lysozyme: A study of the average structures and atomic fluctuations.. J Mol Biol.

[48] Post CB, Karplus M (1986). Does lysozyme follow the lysozyme pathway? an alternative based on dynamic, structural, and stereoelectronic considerations.. J Am Chem Soc.

[49] Jorgensen W, Tirado-Rives J (1988). The OPLS force Field for proteins. Energy minimizations for crystals of cyclic peptides and Crambin.. J Am Chem Soc.

[50] Jorgensen W, Maxwell D, Tirado-Rives J (1996). Development and testing of the OPLS all-atom force field on conformational energetics and properties of organic liquids.. J Am Chem Soc.

[51] Case DA, Cheatham TE, Darden H, Gohlke H, Luo R (2005). The Amber biomolecular simulation programs.. J Computat Chem.

[52] Pearlman DA, Case DA, Caldwell JW, Ross WS, Cheatham TE (1995). AMBER, a package of computer programs for applying molecular mechanics, normal mode analysis, molecular dynamics and free energy calculations to simulate the structural and energetic properties of molecules.. Comp Phys Commun.

[53] Howard BR, Vajdos FF, Li S, Sundquist WI, Hill CP (2003). Structural insights into the catalytic mechanism of cyclophilin A.. Nat Struct Biol.

[54] Northrup SH, Pearl MR, Morgan JD, McCammon JA, Karplus M (1981). Molecular dynamics of ferrocytochrome c: Magnitude and anisotropy of atomic displacements.. J Mol Biol.

[55] Mao B, Pear MR, McCammon JA, Northrup SH (1982). Molecular dynamics of ferrocytochrome c: Anharmonicity of atomic displacements.. Biopolymers.

[56] Ichiye T, Karplus M (1987). Anisotropy and anharmonicity of atomic fluctuations in proteins: Analysis of a molecular dynamics simulation.. Proteins.

[57] Moritsugu K, Miyashita O, Kidera A (2000). Vibrational energy transfer in a protein molecule.. Phys Rev Lett.

[58] Leitner D, Straub JE (2010). Proteins Energy, Heat and Signal Flow..

[59] Ichiye T, Karplus M (1988). Anisotropy and anharmonicity of atomic fluctuations in proteins: Implications for x-ray analysis.. Biochemistry.

[60] Kitao A, Go N (1999). Investigating protein dynamics in collective coordinate space.. Curr Opin Struct Biol.

[61] Brooks B, Karplus M (1983). Harmonic dynamics of proteins: normal modes and fluctuations in bovine pancreatic trypsin inhibitor.. Proc Natl Acad Sci U S A.

[62] Bahar I, Cui Q (2003). Normal Mode Analysis: Theory and Applications to Biological and Chemical Systems..

[63] Bahar I, Rader AJ (2005). Coarse grained normal mode analysis in structural biology.. Curr Opin Struct Biol.

[64] Jolliffe IT (2002). Principal Component Analysis..

[65] Amadei A, Lissen ABM, Berendsen HJC (1993). Essential dynamics of proteins.. Proteins: Struct Funct Genetics.

[66] Materese CK, Goldmon CC, Papoian GA (2008). Hierarchical organization of eglin C native state dynamics is shaped by competing direct and water-mediated interactions.. Proc Natl Acad Sci U S A.

[67] Balsera MA, Wriggers W, Oono Y, Schulten K (1996). Principal component analysis and long time protein dynamics.. J Phys Chem.

[68] Lange OF, Grubmüller H (2006). Can principal components yield a dimension reduced description of protein dynamics on long time scales?. J Phys Chem B.

[69] Altis A, Nguyen P, Hegger R, Stock G (2007). Dihedral angle principal component analysis of molecular dynamics simulations.. J Chem Phys.

[70] Mu Y, Nguyen P, Stock G (2004). Energy landscape of a small peptide revealed by dihedral angle principal component analysis.. Proteins: Struct Funct Bioinform.

[71] Maisuradze GG, Leitner DM (2007). Free energy landscape of a biomolecule in dihedral principal component space: Sampling convergence and correspondence between structures and minima.. Proteins: Struct Funct Bioinform.

[72] Bell AJ, Sejnowski TJ (1995). An information-maximization approach to blind separation and blind deconvolution.. Neural Computation.

[73] Amari S, Cichocki A, Yang HH, Mozer M, Jordan MI, Petsche T (1996). A new learning algorithm for blind signal separation.. Advances in Neural Information Processing Systems 9, NIPS.

[74] Schroder GF (2004). Simulation of Fluorescence Spectroscopy Experiments..

[75] Ferguson AL, Panagiotopoulos AZ, Debenedetti PG, Kevrekidis IG (2010). Systematic determination of order parameters for chain dynamics using diffusion maps.. Proc Natl Acad Sci U S A.

[76] Stamati H, Clementi C, Kavraki L (2010). Application of nonlinear dimensionality reduction to characterize the conformational landscape of small peptides.. Proteins: Struct Funct Bioinform.

[77] Shehu A, Kavraki L, Clementi C (2009). Multiscale characterization of protein conformational ensembles.. Proteins: Struct Funct Bioinform.

[78] Berendsen HJC, Grigera JR, Straatsma TP (1987). The missing term in effective pair potentials.. J Phys Chem.

[79] Berweger CD, van Gunsteren WF, Müller-Plathe F (1995). Force field parametrization by weak coupling. re-engineering SPC water.. Chem Phys Lett.

[80] Caves LS, Evanseck J, Karplus M (1998). Locally accessible conformations of proteins: multiple molecular dynamics simulations of crambin.. Protein Sci.

[81] Shirts MR, Pande VS (2001). Mathematical analysis of coupled parallel simulations.. Phys Rev Lett.

[82] Bowers KJ, Chow E, Xu H, Dror R, Eastwood MP (2006). Scalable algorithms for molecular dynamics simulations on commodity clusters.. SC '06: Proceedings of the 2006 ACM/IEEE conference on Supercomputing.

