# Supplementary figures and images for: Discovering Conformational Sub-States Relevant to Protein Function
Source: PLoS One. 2011 Jan 28;6(1):e15827. doi: 10.1371/journal.pone.0015827 (PMC3030567; doi:10.1371/journal.pone.0015827)

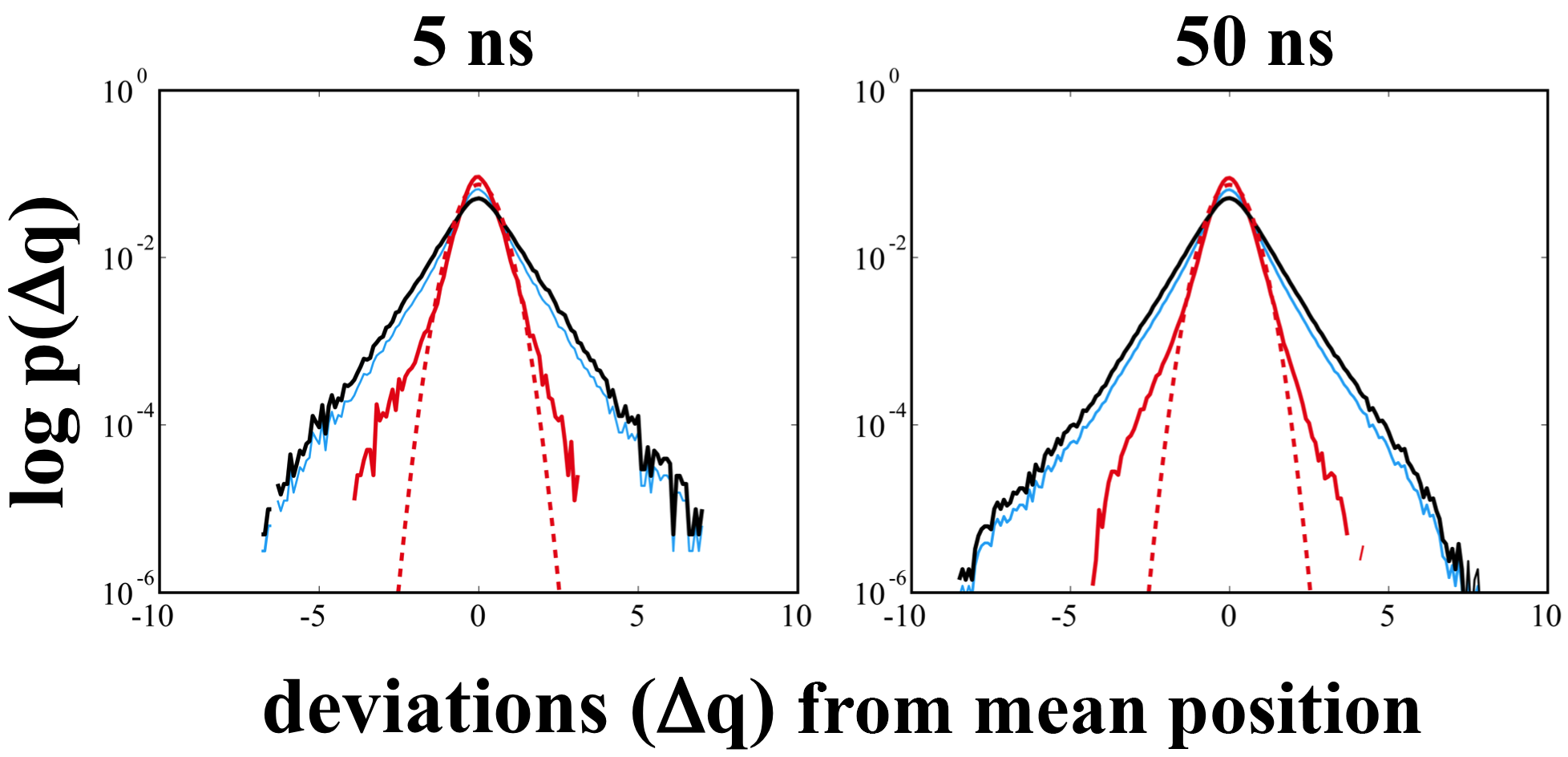

Supplement: Figure S1 — Long-tail distributions at shorter time-scales; side-chains have greater anharmonicity than backbone atoms. Anharmonic distribution of positional deviations (Å) from ubiquitin MD simulations at 5 ns and 50 ns. For each atom, the positional displacement from the time-averaged position was calculated at 50 ps intervals. The same bin size (0.54 Å) was used for all histograms. Distributions correspond to: C (red), Gaussian fit to C (dotted red), side-chains (light blue) and all-atoms (black). The probability distributions of positional deviations [] are plotted in log-scale. (TIF) [file pone.0015827.s001.tif]

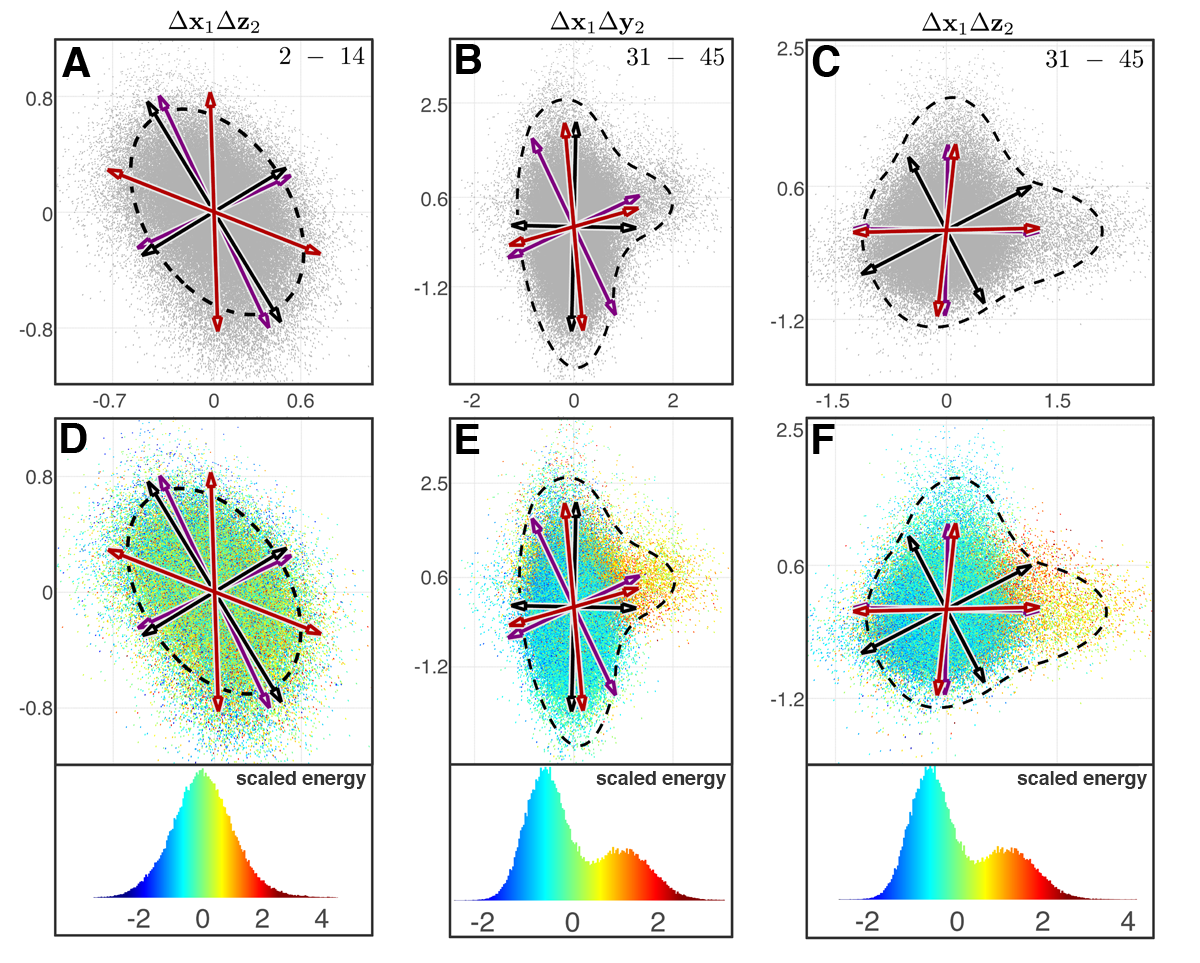

Supplement: Figure S2 — QAA captures intrinsic non-orthogonal directions pointing towards energetically coherent directions in the landscape; QHA and FCA do not. For the ubiquitin simulation (0.5 s), (A) residues 2 and 14 exhibit Gaussian-like fluctuations in the and directions respectively. When pairwise distributions are Gaussian-like, QHA (black) and FCA (purple) basis vectors [34] align well with the intrinsic orientation of the data. Residues 31 and 45 are anharmonic in the (A) and (B) directions, illustrative of modeling challenges for intrinsically non-orthogonal data. QHA (black) and FCA (purple) cannot accurately describe these orientations, whereas QAA (red arrows) align well with the non-orthogonal directions and point towards homogenous energy distributions. (D–F) Distributions identical to (A–C) are colored according to scaled interaction energies (as explained in the main text). QAA basis vectors align with energetically coherent sub-states. In (A–F), dotted lines indicate contours of the non-Gaussian directions in positional fluctuations. Energy distributions are also shown below associated joint distributions; in each the color range is thresholded above and below for visual clarity. All spatial units are in Å. For each residue pair a total of 100,000 conformers were used from the 0.5s simulations. (TIF) [file pone.0015827.s002.tif]

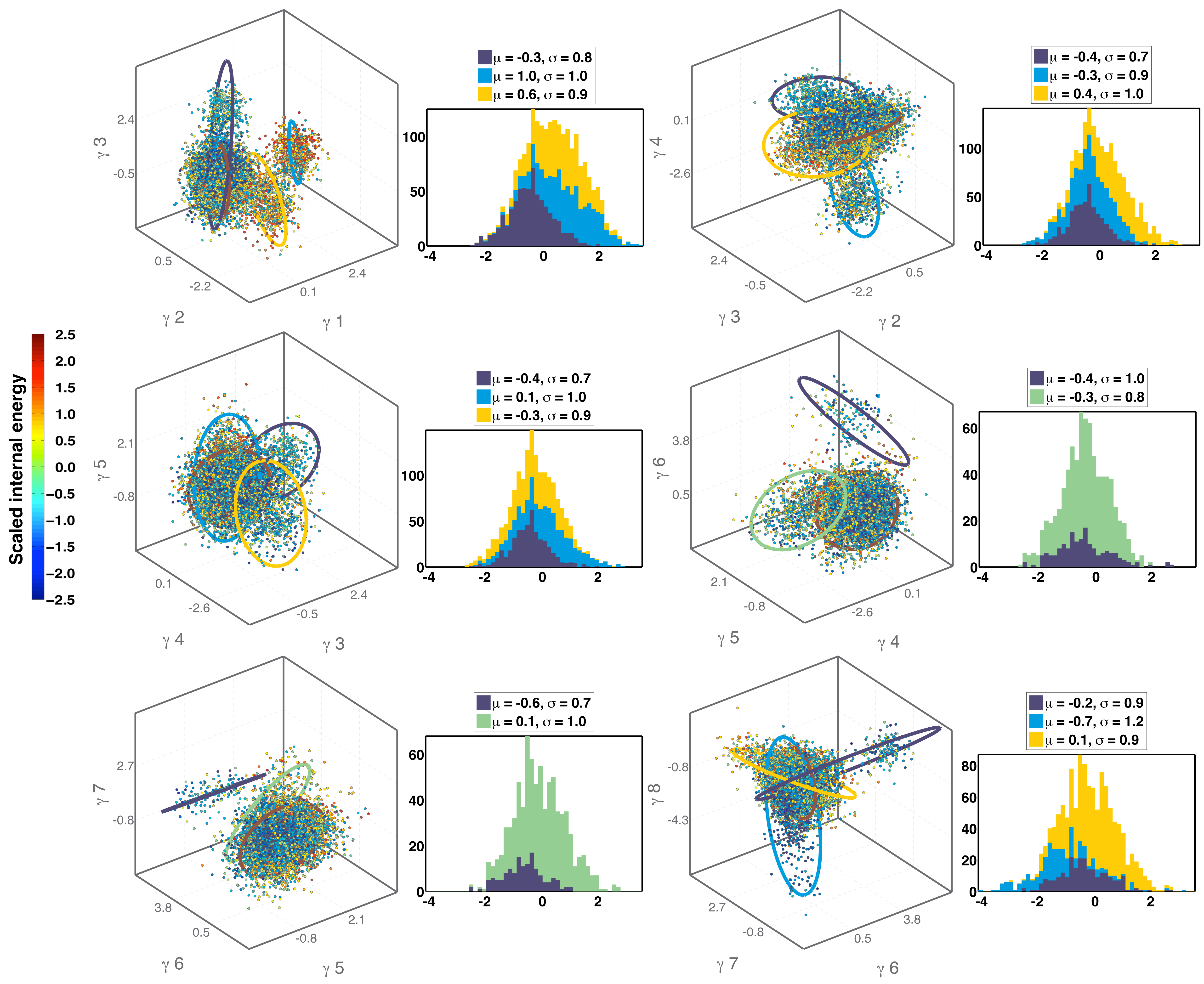

Supplement: Figure S3 — Projections of ubiquitin simulation (0.5 s; 10,000 conformations) onto eight top quasi-anharmonic modes ( ) from QAA illustrate distinct separation in energy distributions. Structures are colored according to scaled (zero mean, unit variance) non-bonded energies, that is, the sum of electrostatic and van der Waals energy terms. Color bins are thresholded at ( - standard deviation). Ellipses indicate clusters determined by mixture of Gaussian (MoG) model [41]. Each cluster is indicated by a colored ellipse whose major and minor axes correspond respectively to the first two principal components of each cluster. Neighboring panels show histograms of energy values within each cluster. Note the colors of the ellipse and histogram match. For each projection, the largest and most energetically heterogenous cluster (brick ellipse) is not included in the histogram to clarify energetic coherency of the remaining (less populated) conformational sub-states. Boxes above the histograms show both the means () and standard deviations () of energy distributions in respective clusters. (TIF) [file pone.0015827.s003.tif]

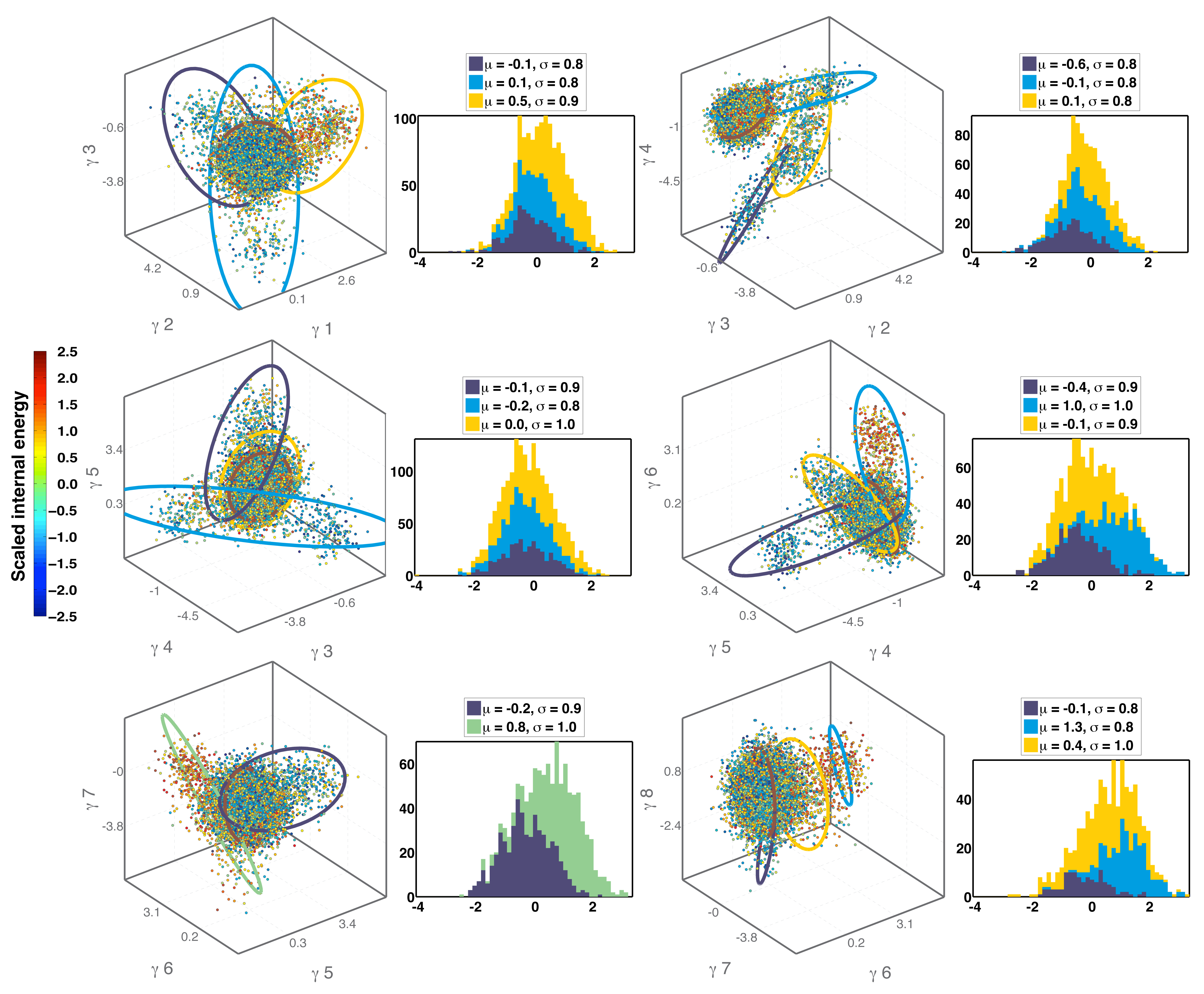

Supplement: Figure S4 — Lysozyme simulation projected onto eight top quasi-anharmonic modes ( ) from QAA illustrate distinct separation in energy distributions. Structures are colored according to scaled internal energies, as explained in the main text. Color bins are thresholded at standard deviations. Ellipses indicate clusters determined by mixture of Gaussian (MoG) model [41]. Each cluster is indicated by a colored ellipse whose major and minor axes correspond respectively to the first two principal components of each cluster. Neighboring panels show histograms of energy values within each cluster. Note the colors of the ellipse and histogram match. For each projection, the largest and most energetically heterogenous cluster (brick ellipse) is not included in the histogram to clarify energetic coherency of the remaining (less populated) conformational sub-states. Boxes above the histograms display means () and standard deviations () of energy distributions in respective clusters. QAA commonly resolves and separates high and low energy sub-states. Projection systems and show clusters (blue ellipses) with mean energies far from global energetic mean ( and respectively versus ), indicating the QAA modes' ability to characterize internal energetics. Compare with Figure 16, where highest resolved cluster mean energy is (FCA). (TIF) [file pone.0015827.s004.tif]

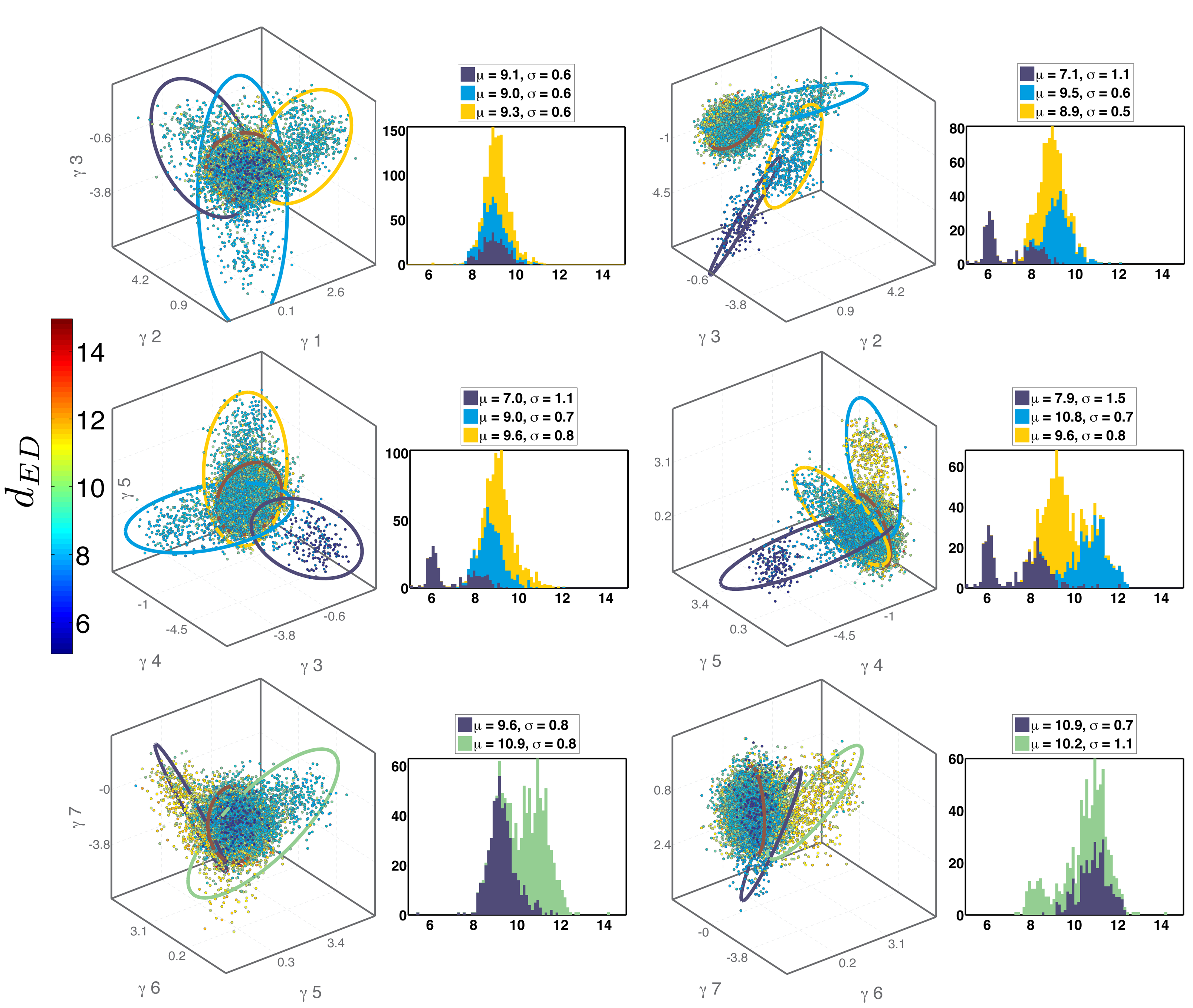

Supplement: Figure S5 — Lysozyme simulation projected onto six QAA coordinate systems. Axis labels correspond to mode indices ranked by fluctuation magnitude, and were chosen sequentially. Structures are colored according to d, the distance between catalytic sites Asp11 and Glu20. Ellipses indicate clusters determined by mixture of Gaussian (MoG) model [41]. Each cluster is indicated by a colored ellipse whose major and minor axes correspond respectively to the first two principal components of each cluster. Neighboring panels show histograms of distances () within each cluster. The colors of the ellipse and histogram match. Note the clear separation between the conformational clusters showing differences in distance (Asp11 to Glu20) distributions. (TIF) [file pone.0015827.s005.tif]

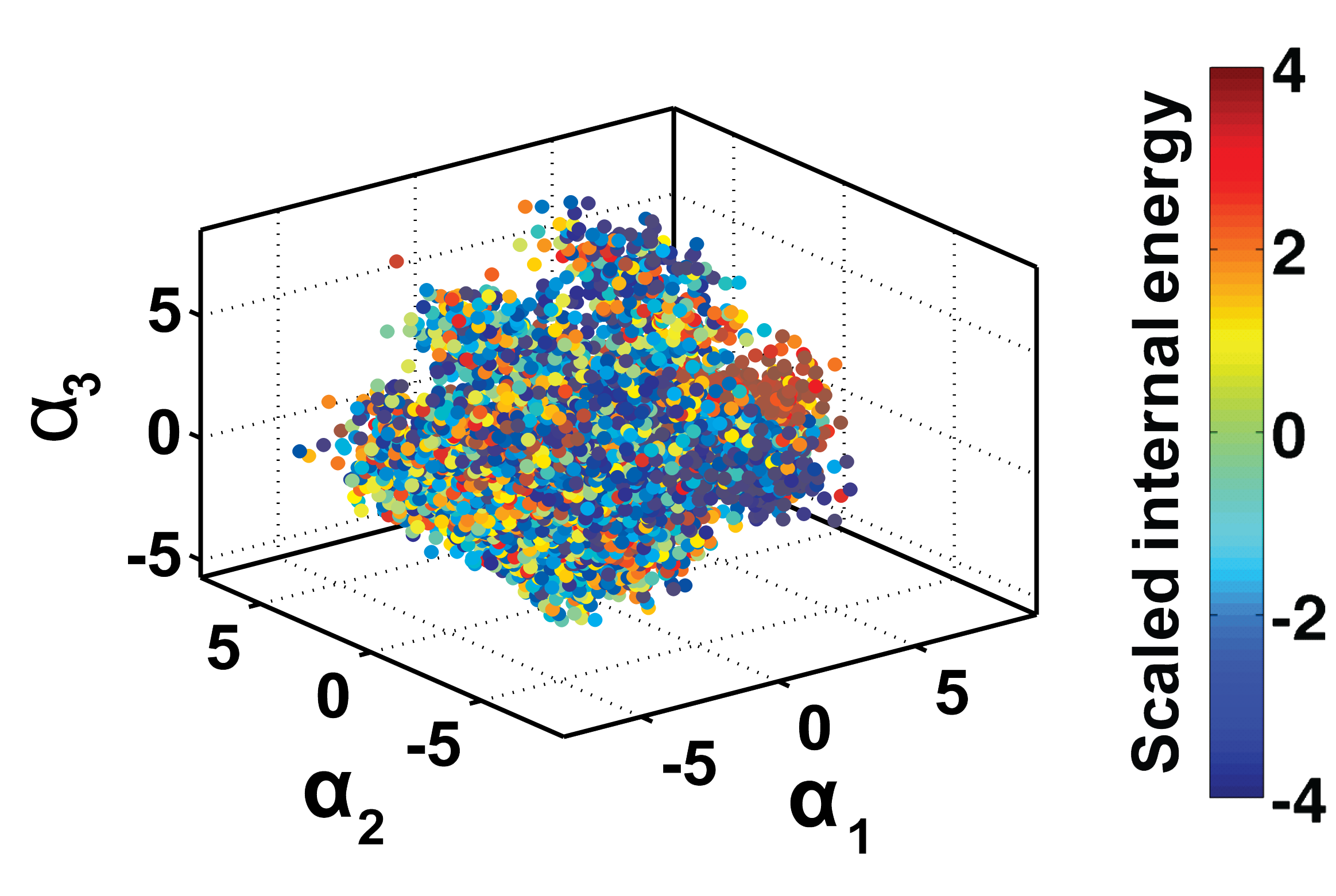

Supplement: Figure S6 — Lack of homogeneity in the internal energy distributions of QHA. For the 0.5 s simulations of ubiquitin (10,000 conformations), the top three basis vectors from QHA ( and ) are depicted here. Projection of each conformation is colored by the scaled internal energy (as described in the main text). Note the apparent lack of clear separation between clusters when compared to QAA (main text, Figure 5). (TIF) [file pone.0015827.s006.tif]

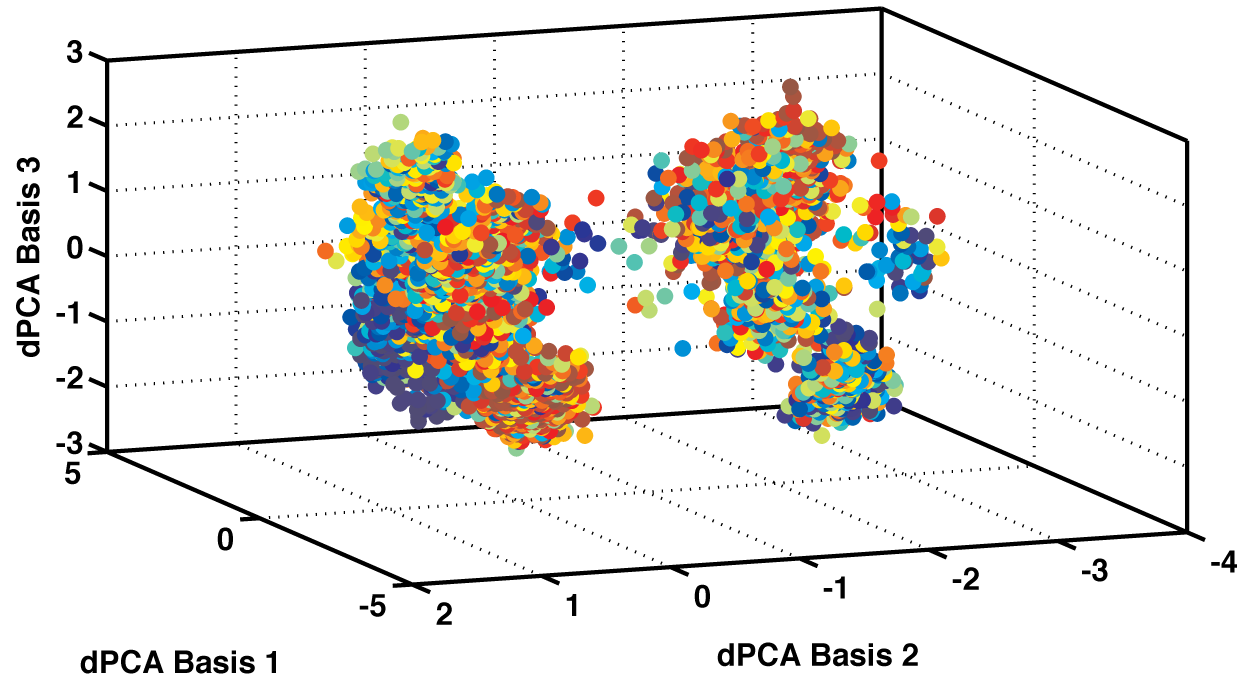

Supplement: Figure S7 — Ubiquitin landscape represented by the first three basis vectors using dihedral PCA [69] from the 0.5 s simulations (10,000 conformations). Projected conformations show the presence of spatial clusters. However, when colored by the scaled internal energy, energetic homogeneity is lacking, unlike in the analogous QAA-based clusters (main text, Figure 5). (TIF) [file pone.0015827.s007.tif]

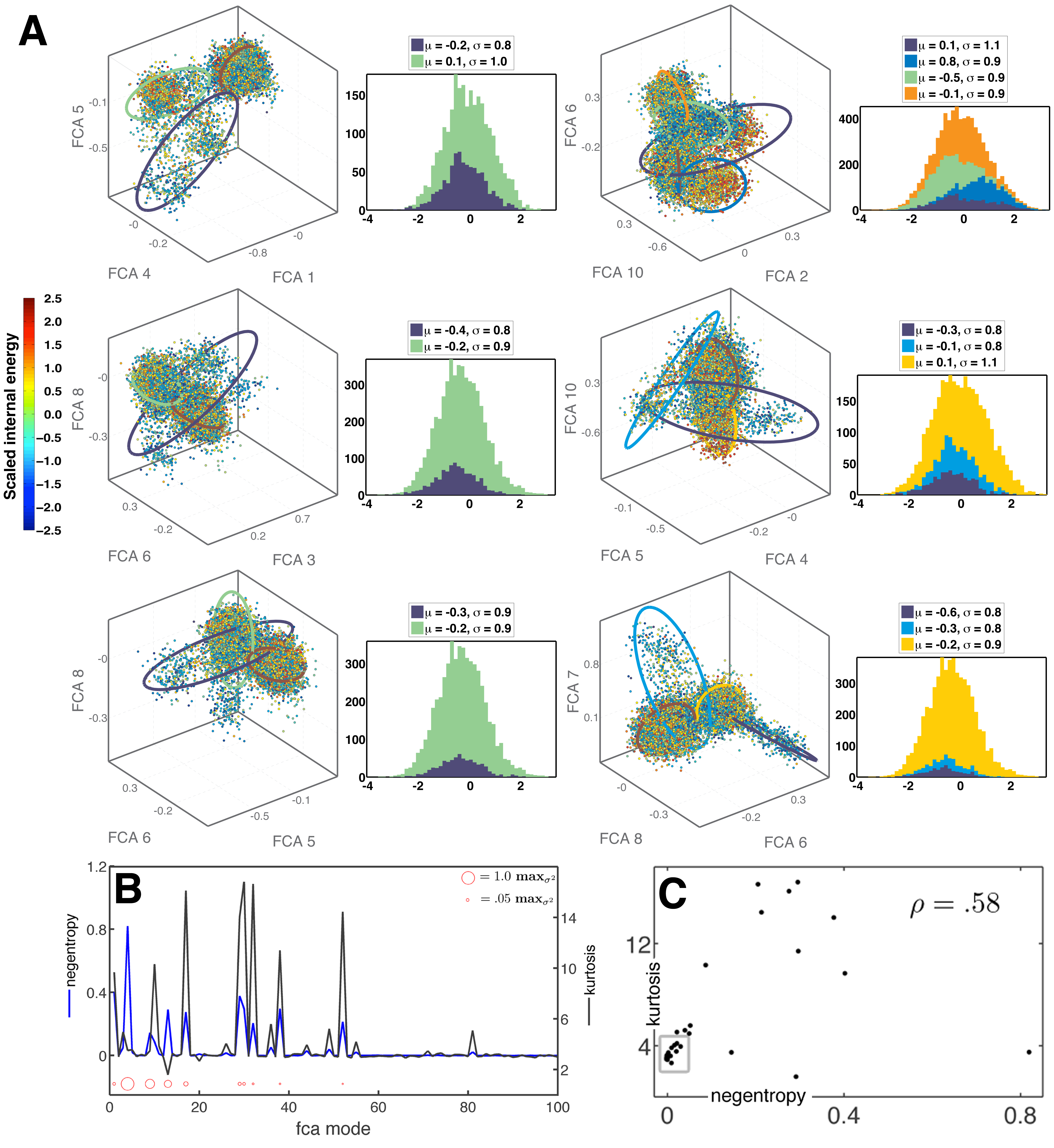

Supplement: Figure S8 — Lysozyme simulations projected onto FCA basis. (A) We follow the protocol used in [34] to consider six projections from FCA (from Figure 12). Axis labels correspond to mode indices ranked by negentropy. Plots and clustering follow the protocol in Figure 12. Excepting FCA and FCA, most projections poorly resolve energetic differences between clusters. (B) Comparison of FCA and negentropy for top 100 FCA modes. Circles indicate the modes selected for the projection coordinates in panel (A) and are sized according to the variance of the associated modes. Note that variance is not a reliable indicator of anharmonicity. (C) Correlation between negentropy and kurtosis for the top 100 FCA modes. Of these modes, 85 display Gaussian statistics ( and negentropy , boxed in grey), suggesting that modes selected by either criteria (kurtosis or negentropy) signify key anharmonic directions. (TIF) [file pone.0015827.s008.tif]

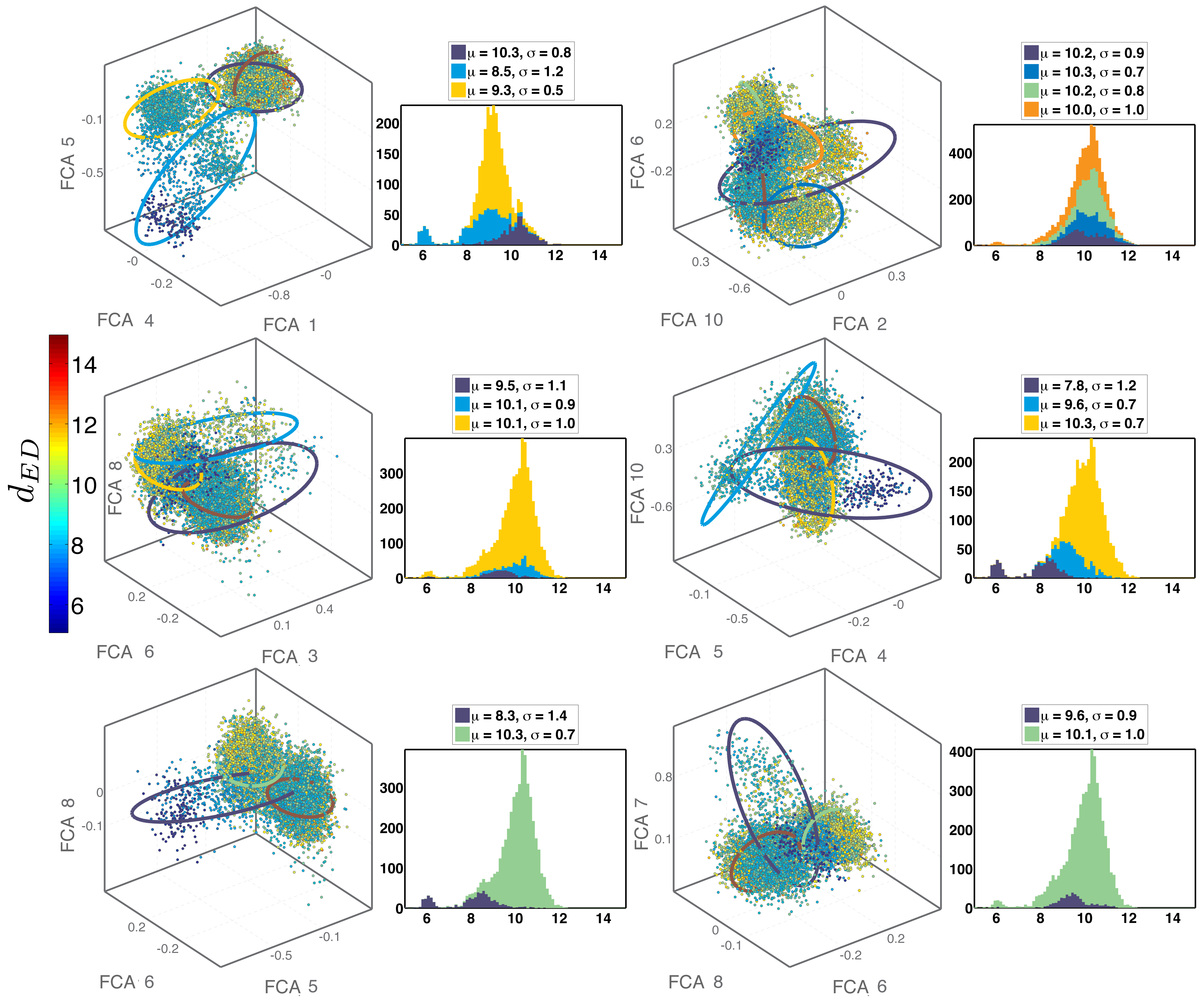

Supplement: Figure S9 — Analysis of Lysozyme simulations using Full Correlation Analysis. (A) Lysozyme simulation projected onto six full correlation analysis (FCA) coordinate systems (Fig. 12) according to procedure in [34]. Axis labels correspond to mode indices after ranking by negentropy. Conformations are colored by the distance between catalytic residues as shown in the previous plot. Observe that the separation between the clusters according to is not as clear as in Figure 13. (TIF) [file pone.0015827.s009.tif]
