# Supplementary material for: Discovering Conformational Sub-States Relevant to Protein Function
Source: PLoS One. 2011 Jan 28;6(1):e15827. doi: 10.1371/journal.pone.0015827 (PMC3030567; doi:10.1371/journal.pone.0015827)
Supplement: Text S1 — Intuition for why QAA finds energetically coherent sub-states (PDF) [file pone.0015827.s013.pdf]

Intuition for why QAA finds energetically coherent sub-states

To understand coupling between different protein regions, we examine the joint positional deviations of atom pairs (Figure S2) and measure how well QAA, full correlation analysis (FCA) [34] and quasi-harmonic analysis (QHA) [36] model the underlying distributions. When the deviations are more Gaussian-like, the QHA basis vectors (black arrows in FigureS2), which maximize variance, align well with the intrinsic orientation of the data. However, when the source distributions combine G^s or G_s, the intrinsic orientations of the data can be non-orthogonal, indicating higher-order correlations. Under these circumstances, QHA fails to capture the intrinsic motions in its sole pursuit of variance.

To provide a biophysical intuition, we also examine the pair-wise distributions in scaled internal energy values (as described in the main text) and plot them as shown in Figure S2 **(D-F)**. The non-orthogonal dependencies give rise to energy bias in the landscape such that moving along these non-orthogonal motion directions results in access to these energetically homogenous sub-states. Observe that QAA basis vectors (red arrows) successfully describe motions that lead towards energetically homogenous sub-states. While FCA also pursues higher-order correlations, it chooses an orthogonal basis representation as illustrated in Figure S2 (purple arrows). Therefore, FCA basis vectors do not align well with energetically coherent directions. Although illustrated for specific pairs of residues, we have exhaustively examined pair-wise residue distributions in ubiquitin to confirm this behavior.
