# Supplementary material for: Discovering Conformational Sub-States Relevant to Protein Function
Source: PLoS One. 2011 Jan 28;6(1):e15827. doi: 10.1371/journal.pone.0015827 (PMC3030567; doi:10.1371/journal.pone.0015827)
Supplement: Text S2 — Comparison of QAA with dihedral PCA (PDF) [file pone.0015827.s014.pdf]

Comparison of QAA with dihedral PCA

Dihedral-PCA (dPCA) uses the internal $\phi - \psi$ angles of the protein backbone as the variables on which PCA is performed [69]. In particular the $\phi - \psi$ angles are converted into a Euclidean representation where each ϕ_i and ψ_i is converted into a 4-tuple: $x_{i-3} = \cos(\phi_i); x_{i-2} = \sin(\phi_i); x_{i-1} = \cos(\psi_i); x_i = \sin(\psi_i)$. For a N residue protein there are a total of $2N$ backbone ϕ and ψ angles. This representation produces a $4P \times 4P$ covariance matrix where $P(= 2N)$ is the total number of ϕ and ψ angles in the protein. The covariance matrix is then diagonalized to produce the corresponding eigenvalues and eigenvectors. The approach is ideally suited in cases where an internal alignment of conformations generated by a simulation is difficult, as in protein folding/unfolding trajectories [69–71].

Our implementation of dPCA for ubiquitin (as shown in Figure S4) also leads to the presence of multiple clusters as observed in previous work on different proteins [69, 71]. However, when the conformations are projected and colored using the scaled internal energies, the clusters in dPCA lack homogeneity. Thus, the ability to find energetically coherent sub-states in QAA makes it unique for this reason.
