# Supplementary material for: Discovering Conformational Sub-States Relevant to Protein Function
Source: PLoS One. 2011 Jan 28;6(1):e15827. doi: 10.1371/journal.pone.0015827 (PMC3030567; doi:10.1371/journal.pone.0015827)
Supplement: Text S3 — Comparing QAA with Full-Correlation Analysis (PDF) [file pone.0015827.s015.pdf]

Comparing QAA with Full-Correlation Analysis

For the lysozyme simulation (120 ns; 12,000 conformations), the anharmonic modes of motion are illustrated in Figure S6 and an analogous plot from FCA is illustrated in Figure S7. Conformers from the simulation are colored according to non-bonded energy, as indicated in the main text. In line with what we observed for ubiquitin, the distributions of scaled internal energies show good separation between sub-states for QAA. For a proper comparison with FCA, we chose the time-scale of lysozyme simulations to be similar to the work presented in [34]. We now examine if the two techniques share any similarity in terms of the description of the landscape.

An interesting comparison between QAA and FCA is presented in Figure S7B. While FCA also pursues a description of anharmonicity and nonlinear correlations using mutual information (MI), QAA pursues κ, kurtosis. A plot showing the overlaps of the kurtosis and MI from the respective methods (for modes extracted with FCA) clearly shows that the information contained in both these techniques are indeed similar. In particular, directions pursued by both QAA and FCA describe the extent of anharmonicity in remarkably similar terms. However, an important distinction arises from the ability of QAA to align with directions that are non-orthogonal. Illustrated on two-dimensional joint positional deviations (Figure S2), we note that FCA directions, although likewise capturing anharmonicity, do not align well with the inherent orientations in the fluctuations. This observation translates to higher dimensions, that is, QAA is capable of detecting anharmonic directions of motion than FCA.

We next compare the ability of QAA with FCA to describe the landscape as a function of the order parameter (d_{ED}) described in the main text of the paper. Note that d_{ED} denotes the distance between catalytically important residues Asp11 and Glu20. As illustrated in Figure S8 and Figure S9, we observe the presence of multiple conformational clusters from both QAA and FCA. However, as the adjoining histogram panels show, QAA is able to better separate the landscape in terms of d_{ED}. This clearly shows QAA's advantage for extracting biophysically relevant low-dimensional representations.
