# Supplementary material for: Discovering Conformational Sub-States Relevant to Protein Function
Source: PLoS One. 2011 Jan 28;6(1):e15827. doi: 10.1371/journal.pone.0015827 (PMC3030567; doi:10.1371/journal.pone.0015827)
Supplement: Text S4 — Movies from QAA for Ubiquitin, Lysozyme and Cyclophilin A (PDF) [file pone.0015827.s016.pdf]

Movies from QAA for Ubiquitin, Lysozyme and Cyclophilin A

Movies S1-S4. For ubiquitin, the movies depict the motions of C^{α} atoms for residues 2-70. Internal motions of ubiquitin are filtered along the 0.5 μs MD simulation along the top-most anharmonic mode (γ_1) at each level (Level 1, Level 2, Level 3 and Level 4) of the hierarchy as illustrated in Figure 5 of the main text. Observe that motions become more local as one descends the hierarchy. The regions showing largest fluctuations are highlighted for visual clarity.

Movies S5-S6. For T4 lysozyme the large-scale motions for Level 1 and Level 2 (shown in Figure 6 of the main text) are shown here. Note that the motions here depict movements of the substrate binding regions very clearly. Also note that the motions in Level 2 show a pronounced opening of the binding cleft, as indicated by an increase in the d_{ED} order parameter (described in the text). The movies also highlight the two sub-domains as well as the relevant motions between the sub-domains that cause the opening and closing of the substrate binding pocket.

Movies S7-S8. For the enzyme cyclophilin A, the movies depict movements of the highlighted regions in Figure 7. QAA modes chosen for our analysis at both Levels 1 and 2 are involved in transiting from the heterogenous conformational well (cluster I) to the transition state (cluster III) indicated by the arrow in Figure 7. The movies highlight key regions in cyclophilin A that are linked to the catalytic activity of the enzyme as observed from previous studies [18, 19]. For visual clarity the substrate is depicted in a stick representation to provide the viewer with a perspective of the catalytic site in cyclophilin A.
